# Association of Epicardial Adipose Tissue Thickness With Heart Rate Variability and Electrocardiographic Markers of Electrical Heterogeneity in Obesity

**DOI:** 10.1111/anec.70238

**Published:** 2026-09-23

**Authors:** Ayhan Coşgun, Hüseyin Ören

**Affiliations:** ^1^ Department of Cardiology Cihanbeyli State Hospital Konya Türkiye; ^2^ Department of Cardiology Ankara City Hospital (Bilkent Campus) Ankara Türkiye

**Keywords:** electrical heterogeneity, epicardial adipose tissue, heart rate variability, obesity, P‐wave dispersion, Tpeak‐to‐Tend interval

## Abstract

**Background:**

Obesity is associated with atrial and ventricular arrhythmias, but the links among excess adiposity, autonomic dysfunction, and electrical heterogeneity remain incompletely defined. We examined whether epicardial adipose tissue (EAT) thickness was associated with heart rate variability (HRV) indices and electrocardiographic markers of electrical heterogeneity in adults with obesity.

**Methods:**

In this cross‐sectional study conducted between January 2023 and June 2025, 129 adults with obesity (BMI ≥ 30 kg/m^2^) and 156 normal‐weight controls (BMI < 25 kg/m^2^) with broadly similar group‐level age and sex distributions were evaluated. EAT thickness was measured by transthoracic echocardiography. Autonomic modulation was assessed by 24‐h HRV. Electrocardiograms were analyzed for P‐wave dispersion (PWD), Tpeak‐to‐Tend (Tp‐e) interval, Tp‐e dispersion, and Tp‐e/QT ratio. The prespecified primary endpoints were PWD and Tp‐e interval.

**Results:**

Compared with controls, individuals with obesity had greater EAT thickness (7.2 ± 1.1 vs. 4.2 ± 2.1 mm), higher PWD (30.9 ± 5.8 vs. 17.5 ± 2.4 ms), and longer Tp‐e interval (96.2 ± 8.8 vs. 83.5 ± 7.4 ms; all *p* < 0.001). Between‐group differences were 13.6 ms for PWD (95% CI 12.5–14.7) and 12.7 ms for Tp‐e (95% CI 10.8–14.6). Obesity was also associated with lower SDNN and RMSSD, higher LF/HF ratio, and higher Tp‐e dispersion, whereas Tp‐e/QT showed only a nominal exploratory difference. In multivariable linear regression, EAT thickness remained independently associated with PWD (standardized β = 0.38, *p* < 0.001) and Tp‐e interval (standardized β = 0.35, *p* < 0.001).

**Conclusion:**

Greater EAT thickness in obesity was associated with an unfavorable HRV profile and increased atrial and ventricular electrical heterogeneity. These findings are associative and should not be interpreted as clinical rhythm‐risk stratification.

## Graphical abstract

Epicardial adipose tissue thickness was evaluated in adults with obesity and normal‐weight controls using transthoracic echocardiography, 24‐h heart rate variability analysis, and electrocardiographic markers of electrical heterogeneity. Individuals with obesity showed a greater EAT thickness, a higher P‐wave dispersion, a longer Tp‐e interval, a higher Tp‐e dispersion, and an unfavorable HRV profile characterized by lower SDNN and RMSSD and a higher LF/HF ratio. In multivariable linear regression, EAT thickness remained independently associated with both P‐wave dispersion and Tp‐e interval. These findings suggest that increased EAT thickness may reflect an adverse autonomic–electrophysiological phenotype in obesity. Because of the retrospective cross‐sectional design and selected symptomatic outpatient cohort, the findings should be interpreted as associative rather than predictive or diagnostic.

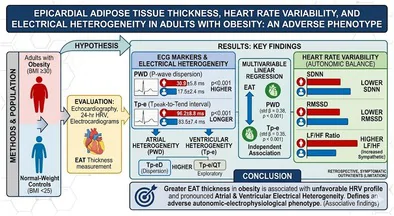

## 1 Introduction

Obesity is associated with a higher burden of both atrial and ventricular arrhythmias (Ng et al. 2014; World NCD Risk Factor Collaboration (NCD‐RisC) 2024). Yet the mechanisms linking excess adiposity to electrical heterogeneity remain incompletely understood. Among the pathways implicated in this relationship, autonomic dysregulation appears to be especially important. In obesity, sympathetic activity tends to increase, whereas parasympathetic modulation declines, a pattern that is commonly reflected by impaired heart rate variability (HRV) (Grassi et al. 2019; La Rovere et al. 1998; Rossi et al. 2015). This autonomic imbalance has been associated with adverse cardiovascular outcomes, including arrhythmic events and sudden cardiac death (Grassi et al. 2019; La Rovere et al. 1998; Rossi et al. 2015).

Epicardial adipose tissue (EAT) has attracted increasing attention in this context. Located between the myocardium and the visceral pericardium, EAT is not simply an inert fat depot. Rather, it is a biologically active visceral tissue capable of releasing proinflammatory cytokines, adipokines, and vasoactive mediators that may influence myocardial structure and electrophysiology (Iacobellis and Bianco 2011; Mahabadi et al. 2013). Because of its direct anatomical proximity to the heart, EAT may be particularly relevant to obesity‐related electrical remodeling.

Previous studies have linked greater EAT burden to both atrial and ventricular arrhythmias (Al Chekakie et al. 2010; Batal et al. 2010; Drossos et al. 2014). Proposed mechanisms include local inflammation, myocardial remodeling, autonomic dysregulation, and increased electrical heterogeneity (Iacobellis and Bianco 2011; Mahabadi et al. 2013; Al Chekakie et al. 2010; Batal et al. 2010; Drossos et al. 2014; Nasri et al. 2018). From an electrophysiological standpoint, electrocardiographic markers such as P‐wave dispersion (PWD), the Tpeak‐to‐Tend (Tp‐e) interval, and the Tp‐e/QT ratio are widely used to reflect atrial and ventricular electrical heterogeneity and have been associated with arrhythmic susceptibility (Dilaveris and Gialafos 2001; Goldman et al. 2023; Task Force of the European Society of Cardiology and the North American Society of Pacing and Electrophysiology 1996). PWD is generally regarded as a marker of heterogeneous atrial conduction, whereas Tp‐e and related repolarization indices reflect heterogeneity of ventricular repolarization (Dilaveris and Gialafos 2001; Goldman et al. 2023; Task Force of the European Society of Cardiology and the North American Society of Pacing and Electrophysiology 1996).

HRV provides a complementary, noninvasive assessment of cardiac autonomic modulation (Task Force of the European Society of Cardiology and the North American Society of Pacing and Electrophysiology 1996; Grassi et al. 2020; Wong et al. 2011). Because HRV is not a single parameter, reduced HRV should be interpreted according to the specific metric examined. Among time‐domain measures, lower SDNN reflects reduced overall heart rate variability, whereas lower RMSSD predominantly indicates diminished short‐term, vagally mediated modulation. Frequency‐domain measures characterize the spectral distribution of variability; LF and HF power reflect different but overlapping components of autonomic modulation, while the LF/HF ratio describes their relative distribution and should not be regarded as a direct measure of sympathovagal balance. Lower time‐domain HRV and altered frequency‐domain profiles have been associated with increased arrhythmic vulnerability across various clinical settings (Task Force of the European Society of Cardiology and the North American Society of Pacing and Electrophysiology 1996; Grassi et al. 2020; Wong et al. 2011). Accordingly, evaluating HRV alongside ECG‐based markers of electrical heterogeneity may provide a more integrated characterization of the cardiovascular phenotype associated with obesity (Grassi et al. 2020; Wong et al. 2011).

Although the relationships among obesity, EAT, autonomic regulation, and arrhythmia‐related ECG markers have increasingly been explored, studies assessing these components together remain limited. In particular, the association of EAT thickness with both HRV indices and ECG markers of electrical heterogeneity has not been clearly defined in adults with obesity (Chen et al. 2022; Wong et al. 2017; Nerlekar and Ha 2025; Patel and Haldar 2022; Nakamori et al. 2018). In the present study, we evaluated EAT thickness, HRV indices, and ECG‐derived markers of electrical heterogeneity in adults with obesity and in normal‐weight controls with similar age and sex distributions. We hypothesized that greater EAT thickness would be associated with less favorable HRV indices and with higher PWD and Tp‐e values (Rossi et al. 2015; Iacobellis and Bianco 2011; Mahabadi et al. 2013; Al Chekakie et al. 2010; Dilaveris and Gialafos 2001; Goldman et al. 2023; Task Force of the European Society of Cardiology and the North American Society of Pacing and Electrophysiology 1996; Patel and Haldar 2022; Nakamori et al. 2018; Shamloo et al. 2019). Our primary aim was to characterize the autonomic and electrophysiological alterations associated with obesity and to determine whether the associations of EAT thickness with the primary electrical heterogeneity markers remained significant after adjustment for relevant clinical and metabolic variables (Al Chekakie et al. 2010; Batal et al. 2010; Drossos et al. 2014; Nasri et al. 2018; Shamloo et al. 2019; Anagnostopoulos et al. 2023; Conte et al. 2022; Bertaso et al. 2013; Iacobellis et al. 2005).

## 2 Materials and Methods

### 2.1 Study Design and Ethics

This was a single‐center, retrospective cross‐sectional study conducted at Ankara City Hospital (Bilkent Campus). The study population was identified through retrospective screening of hospital records obtained between January 2023 and June 2025. Clinical, laboratory, electrocardiographic, Holter, and echocardiographic data had been acquired during routine outpatient evaluation and were analyzed retrospectively for the purposes of this study.

The study protocol was approved by the Ethics Committee of Ankara City Hospital (approval no. TABED 2‐25‐1652/25‐11‐2025) and the study was conducted in accordance with the principles of the Declaration of Helsinki (2013 revision). The requirement for individual informed consent was waived because of the retrospective design.

### 2.2 Study Population and Participant Selection

Participants were identified through retrospective review of cardiology outpatient clinic records at Ankara City Hospital (Bilkent Campus). All included individuals had undergone routine outpatient cardiologic evaluation during the study period. Twenty‐four‐hour ambulatory Holter monitoring had been performed because of palpitations, whereas resting 12‐lead electrocardiography and transthoracic echocardiography were obtained as part of standard clinical assessment. Resting ECG, transthoracic echocardiography, initiation of 24‐h Holter monitoring, fasting laboratory testing, and clinical and anthropometric measurements, including blood pressure, body weight, height, and waist circumference, were all performed on the same day. Only participants in sinus rhythm at the time of ECG acquisition and Holter analysis were eligible.

**FIGURE 1:**
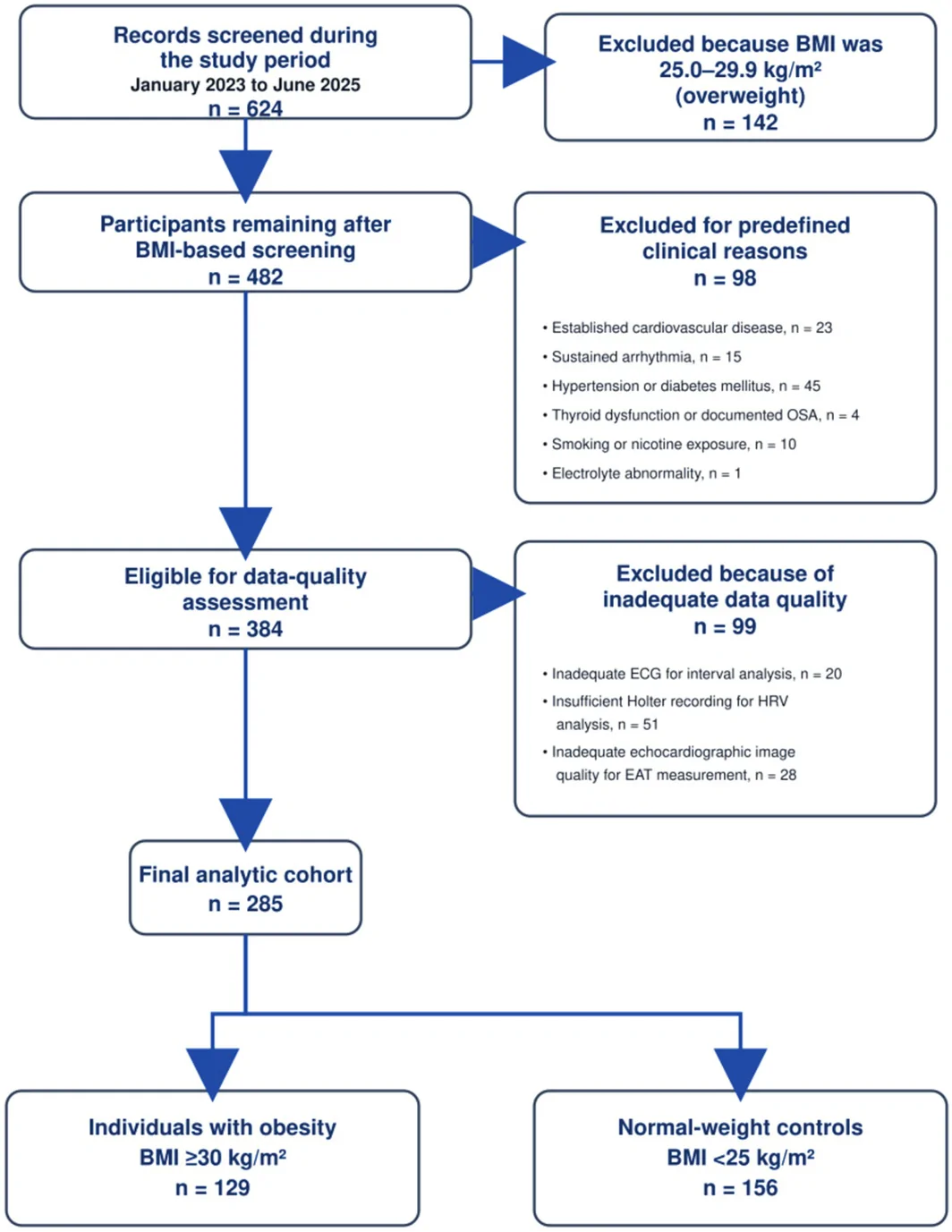
Study flow diagram. Flow diagram showing participant selection for the final analytic cohort. A total of 624 records were screened between January 2023 and June 2025. Individuals with overweight status (BMI 25.0–29.9 kg/m²) were excluded a priori to maximize phenotypic separation. After subsequent clinical screening and data‐quality assessment, 285 participants were included in the final analysis, comprising 129 individuals with obesity and 156 normal‐weight controls. ECG indicates electrocardiogram; HRV, heart rate variability; EAT, epicardial adipose tissue; BMI, body mass index.

Eligibility required the availability of anthropometric measurements, fasting laboratory data, a resting ECG of sufficient quality for interval analysis, transthoracic echocardiography with measurable epicardial adipose tissue (EAT), and 24‐h Holter recordings of sufficient quality for heart rate variability analysis. A total of 624 records were screened during the study period. Because the study was designed to compare two clearly separated adiposity phenotypes rather than to evaluate a graded association across the full BMI spectrum, individuals with overweight status (BMI 25.0–29.9 kg/m^2^; *n* = 142) were excluded a priori. This approach enhanced the contrast between the normal‐weight and obesity groups but precluded evaluation of participants with an intermediate adiposity phenotype. The remaining 482 participants underwent further eligibility assessment (Figure 1).

Exclusion criteria were prespecified to minimize major clinical, biochemical, behavioral, and treatment‐related confounders that could influence cardiac autonomic function, ventricular repolarization, or echocardiographic assessment of EAT. Participants were excluded if they had established cardiovascular disease, including coronary artery disease, previous myocardial infarction, prior coronary revascularization, heart failure, cardiomyopathy, significant valvular heart disease, or congenital heart disease. Individuals with atrial fibrillation or other sustained arrhythmias were also excluded because such rhythm disorders could compromise the validity of ECG‐ and Holter‐based analyses. Additional exclusion criteria included hypertension, diabetes mellitus, thyroid dysfunction, and documented obstructive sleep apnea, given their known effects on autonomic tone, myocardial electrical properties, and cardiac structure. To minimize behavioral and physiological confounding, individuals with current smoking, nicotine use, regular passive smoke exposure, pregnancy, or lactation were excluded. Participants with abnormal same‐day serum electrolyte values were not included because electrolyte disturbances may affect repolarization indices. Use of medications known to influence cardiac autonomic regulation or ventricular repolarization also led to exclusion, including beta‐blockers, antiarrhythmic drugs, antihypertensive agents, statins, antidepressants, sympathomimetic agents, and other QT‐prolonging drugs. Of the 482 participants undergoing eligibility assessment, 98 were excluded for these predefined clinical reasons.

The remaining 384 participants proceeded to data‐quality assessment. At this stage, 99 participants were excluded because of inadequate data quality, including insufficient ECG quality for interval analysis (*n* = 20), inadequate Holter recordings for reliable heart rate variability analysis (*n* = 51), and inadequate echocardiographic image quality for accurate EAT measurement (*n* = 28). The final analytic cohort therefore consisted of 285 adults, including 129 individuals with obesity (BMI ≥ 30 kg/m^2^) and 156 normal‐weight controls (BMI < 25 kg/m^2^). The control group was selected from the same cardiology outpatient population and consisted of normal‐weight individuals with similar age and sex distributions who had also presented with palpitations and had undergone the same diagnostic evaluation. Accordingly, the control group should be interpreted as a clinic‐based comparison group rather than a healthy volunteer population (Figure 1).

### 2.3 Study Endpoints

The study was designed with two prespecified co‐primary electrophysiological endpoints: P‐wave dispersion (PWD) and the Tpeak‐to‐Tend (Tp‐e) interval. The prespecified secondary autonomic endpoints were SDNN, RMSSD, and the LF/HF ratio. Additional electrocardiographic and autonomic variables, including Tp‐e dispersion (Tp‐eD), Tp‐e/QT ratio, QT, QTc‐B, QTc‐F, basal ECG heart rate, P_max_, and P_min_, were evaluated as exploratory measures. These exploratory variables were intended to provide supportive phenotypic information and were interpreted as descriptive and hypothesis‐generating rather than confirmatory outcomes.

### 2.4 Clinical, Anthropometric, and Laboratory Assessment

Body weight and height were measured with participants wearing light clothing and no shoes, and BMI was calculated as weight in kilograms divided by height in meters squared. Waist circumference was measured at the midpoint between the lower margin of the last palpable rib and the iliac crest at the end of normal expiration using a non‐elastic tape. Each anthropometric variable was measured twice, and the mean value was used for analysis.

Resting blood pressure was measured in a quiet, temperature‐controlled room after at least 10 min of seated rest using a validated automated sphygmomanometer. Two consecutive readings were obtained and averaged. If the difference between the two measurements exceeded 5 mmHg, a third measurement was performed, and the closest two values were averaged.

After an overnight fast of at least 12 h, venous blood samples were collected under standardized conditions. Fasting glucose, total cholesterol, low‐density lipoprotein cholesterol (LDL‐C), high‐density lipoprotein cholesterol (HDL‐C), and triglycerides were measured in the hospital's central laboratory using routine enzymatic colorimetric methods.

### 2.5 Echocardiographic Assessment of Epicardial Adipose Tissue

Transthoracic echocardiography was performed using a Vivid 7 system (GE Healthcare, USA) equipped with a 2.5–3.5 MHz phased‐array transducer. Epicardial adipose tissue thickness was measured according to the echocardiographic method described by Iacobellis et al. (Iacobellis and Bianco 2011). EAT was defined as the echo‐free space between the outer wall of the myocardium and the visceral pericardium and was measured in the parasternal long‐axis view at end‐systole. Measurements were obtained over three consecutive cardiac cycles, and the mean value was used for analysis.

For study purposes, archived echocardiographic images were reviewed offline, and EAT thickness was measured by an experienced cardiologist blinded to the clinical, laboratory, and electrocardiographic data. Images were digitally stored for offline review. To assess reproducibility, EAT thickness was re‐measured in a random subset of 30 participants by the same observer and by a second blinded observer.

### 2.6 Electrocardiographic Acquisition and Analysis

Standard resting 12‐lead electrocardiograms were recorded in the supine position after at least 10 min of rest in a quiet environment. ECGs were acquired during spontaneous breathing using a calibrated recording system at a paper speed of 50 mm/s and an amplitude of 20 mm/mV. Digital ECGs were analyzed offline using magnified images and electronic calipers. All interval measurements were reported in milliseconds.

Directly measured ECG intervals included P‐wave duration in each eligible lead, the QT interval, and the Tpeak‐to‐Tend (Tp‐e) interval. P_max_ and P_min_ were directly determined as the longest and shortest lead‐specific P‐wave durations, respectively. P‐wave dispersion (PWD), Tp‐e dispersion (Tp‐eD), the Tp‐e/QT ratio, and the Bazett‐ and Fridericia‐corrected QT intervals were subsequently calculated from these directly measured values.

Given the recognized interobserver variability in manual QT measurement and subsequent QTc assessment (Viskin et al. 2005), all directly measured ECG intervals were independently assessed by two cardiologists blinded to the study group and echocardiographic findings. When the measurements of a directly measured interval differed by more than 5 ms between the two observers, the relevant interval was jointly reassessed, and the final value was determined by consensus. The 5‐ms agreement criterion was applied to lead‐specific P‐wave durations, including the measurements determining P_max_ and P_min_, and to the QT and Tp‐e intervals. It was not applied separately to the derived parameters PWD, Tp‐eD, Tp‐e/QT, QTc‐B, or QTc‐F.

#### 2.6.1 P‐Wave Analysis

P‐wave onset was defined as the point at which the first atrial deflection departed from the isoelectric line, and P‐wave offset as the point at which the waveform returned to baseline. P‐wave duration was measured from P‐wave onset to P‐wave offset in each eligible lead. The longest and shortest P‐wave durations across the 12 leads were defined as P_max_ and P_min_, respectively, and PWD was calculated as P_max_−P_min_. Leads in which P‐wave onset or offset could not be clearly identified were excluded from the analysis.

#### 2.6.2 Ventricular Repolarization Analysis

The Tp‐e interval was defined as the interval from the peak of the T wave to the end of the T wave. Tp‐e measurements were prespecified in the precordial leads V2–V6. This lead set was selected because Tp‐e is lead‐dependent, and comparative 12‐lead evidence indicates that maximal Tp‐e values are most frequently captured in the precordial leads, particularly V2–V4 under resting conditions (Ruedisueli et al. 2022). Accordingly, leads V2–V6 was used to provide broader precordial coverage while maintaining a standardized lead‐selection protocol. Lead V1 was not included because normal T‐wave polarity in this lead is physiologically variable in adults and may be upright or inverted, which may complicate reproducible identification of the T‐wave peak and end (Rautaharju et al. 2009). Limb leads were excluded to maintain a standardized precordial‐lead assessment and because maximal Tp‐e values are less frequently captured in the limb leads than in the precordial leads (Ruedisueli et al. 2022). Within V2–V6, leads with low‐amplitude, biphasic, or poorly defined T waves were excluded from measurement. Measurements in each eligible lead were averaged over three consecutive beats, and the mean of the acceptable lead‐specific measurements was used as the Tp‐e value for analysis. The maximum and minimum Tp‐e values were identified across the eligible V2–V6 leads, and Tp‐e dispersion (Tp‐eD) was calculated as their difference.

The QT interval was measured in the same eligible precordial leads used for Tp‐e analysis (V2–V6), from the onset of the QRS complex to the end of the T wave. The end of the T wave was defined using the tangent method as the point at which the tangent to the terminal downslope of the T wave intersected the isoelectric line; in the presence of a U wave, it was defined as the nadir between the T and U waves. Leads with low‐amplitude, biphasic, or poorly defined T waves were excluded. In each eligible lead, QT and Tp‐e intervals were measured over three consecutive sinus beats and averaged to obtain lead‐specific values. Participant‐level QT and Tp‐e values were subsequently calculated as the means of the acceptable lead‐specific values across V2–V6. The Tp‐e/QT ratio was calculated by dividing the participant‐level mean Tp‐e interval by the participant‐level mean QT interval, rather than by averaging individual lead‐specific ratios. Heart rate‐corrected QT intervals were calculated from the participant‐level QT value using both Bazett's formula (QTc‐B = QT/√RR) and Fridericia's formula (QTc‐F = QT/RR^1^ᐟ^3^), where RR represents the mean preceding R–R interval obtained from the same three beats and expressed in seconds.

Basal ECG heart rate was generated automatically by the digital ECG recording system and was not manually measured.

### 2.7 Measurement Reproducibility

Measurement reproducibility was assessed using intraclass correlation coefficients (ICCs) derived from a two‐way random‐effects model with absolute agreement. For EAT thickness, the intraobserver ICC was 0.92 (95% confidence interval [CI] 0.88–0.95) and the interobserver ICC was 0.90 (95% CI 0.85–0.94). Parameter‐specific reproducibility estimates are presented in Table S6. Based on the point estimates, intraobserver reliability was good to excellent, with ICCs ranging from 0.82 for Tp‐eD to 0.94 for the QT interval, whereas interobserver reliability was good, with ICCs ranging from 0.78 for PWD and Tp‐eD to 0.90 for EAT thickness and the QT interval. The derived QTc‐B, QTc‐F, and Tp‐e/QT measures also demonstrated good reproducibility, with intraobserver ICCs ranging from 0.87 to 0.89 and interobserver ICCs ranging from 0.85 to 0.86. Confidence intervals were wider for the dispersion measures, particularly PWD and Tp‐eD, indicating greater uncertainty for these parameters.

To assess intraobserver reproducibility, EAT thickness and all electrocardiographic parameters listed in Table S6 were remeasured by the primary observer in a randomly selected subset of 30 participants after a two‐week interval, with the observer blinded to the initial measurements. For interobserver reproducibility, the same recordings were independently evaluated by a second observer who was blinded to the measurements of the primary observer. ICCs were calculated from the paired repeated measurements obtained before consensus adjudication.

### 2.8 Holter Monitoring and Heart Rate Variability Analysis

All participants underwent 24‐h ambulatory Holter ECG monitoring using a three‐channel digital recorder (DMS 300–7, USA) with a sampling frequency of 1000 Hz. Participants were instructed to maintain their usual daily routine but to avoid strenuous exercise and caffeine during monitoring. Resting ECG, transthoracic echocardiography, fasting laboratory testing, and initiation of 24‐h Holter monitoring were performed on the same day.

All Holter recordings were visually inspected for signal quality before analysis. Only recordings with at least 22 h of analyzable data and < 5% artifact contamination were accepted. Recordings containing excessive noise or more than 5% non‐sinus beats or artifacts were excluded. Ectopic beats and artifacts were handled using adaptive filtering and local interpolation before HRV calculation.

HRV analysis was performed in accordance with the standards of the Task Force of the European Society of Cardiology and the North American Society of Pacing and Electrophysiology (Task Force of the European Society of Cardiology and the North American Society of Pacing and Electrophysiology 1996). Time‐domain indices included the standard deviation of all normal‐to‐normal intervals (SDNN) and the root mean square of successive differences (RMSSD). For frequency‐domain analysis, power spectral density was estimated using a fast Fourier transform (FFT) after linear detrending and Hanning windowing. Low‐frequency (LF, 0.04–0.15 Hz) and high‐frequency (HF, 0.15–0.40 Hz) components were derived, and the LF/HF ratio was calculated as a relative spectral distribution.

For spectral analysis, artifact‐free 5‐min segments were selected from predefined daytime (10:00–20:00) and nighttime (00:00–06:00) periods and averaged for analysis. HRV processing was performed using the CardioScan HRV Analyzer within the DMS Holter system and was verified by an investigator blinded to the study group.

The recording‐level quality criteria were applied because SDNN and RMSSD were calculated from the entire 24‐h normal‐to‐normal interval series rather than solely from selected 5‐min segments. When the overall burden of artifacts or non‐sinus beats exceeds 5%, extensive beat removal and interpolation may materially affect time‐domain HRV estimates. The artifact‐free 5‐min segments were used only for frequency‐domain analysis. Accordingly, the recording‐level threshold was retained to ensure reliable time‐domain measurements and a consistent analytic cohort across the prespecified HRV endpoints.

All 51 participants excluded from the primary Holter analysis nevertheless had at least one artifact‐free 5‐min segment available within each of the prespecified daytime and nighttime periods. Accordingly, all 51 participants were included in the expanded segment‐based sensitivity analysis of the LF/HF ratio, increasing the sample size from 285 to 336 participants.

### 2.9 Statistical Analysis

All statistical analyses were performed using IBM SPSS Statistics for Windows, version 25.0 (IBM Corp., Armonk, NY, USA). The distribution of continuous variables was assessed using the Shapiro–Wilk test together with visual inspection of Q–Q plots. Homogeneity of variance was evaluated using Levene's test.

Normally distributed continuous variables are presented as mean ± standard deviation (SD), whereas non‐normally distributed variables are presented as median (interquartile range). Categorical variables are presented as counts and percentages. Between‐group comparisons were performed using Welch's *t*‐test for continuous variables and the Mann–Whitney U test for non‐normally distributed variables. Categorical variables were compared using the chi‐square test or Fisher's exact test, as appropriate.

Associations between EAT thickness and electrocardiographic or HRV variables were examined using Pearson or Spearman correlation analysis, as appropriate according to variable distribution. Multivariable linear regression models were then constructed to evaluate the associations of EAT thickness with the co‐primary endpoints (PWD and Tp‐e) after adjustment for clinically relevant covariates selected a priori, including age, sex, body mass index (BMI), systolic blood pressure, and high‐density lipoprotein cholesterol (HDL‐C). HDL‐C was selected a priori as the lipid marker most relevant to the cardiometabolic profile and least redundant with the remaining covariates. To assess multicollinearity, variance inflation factors (VIFs) were calculated, with VIF < 3 considered acceptable; all VIF values in the final models were < 3.

### 2.10 Multiplicity and Endpoint Hierarchy

The study specified two co‐primary electrophysiological endpoints, P‐wave dispersion (PWD) and the Tpeak‐to‐Tend (Tp‐e) interval. For these endpoints, a two‐sided *p*‐value < 0.05 was considered statistically significant. The prespecified secondary autonomic endpoints were SDNN, RMSSD, and the LF/HF ratio; for these three endpoints, Bonferroni correction was applied, with statistical significance defined as *p* < 0.0167. All other electrocardiographic and autonomic variables, including basal ECG heart rate, QT, QTc‐B, QTc‐F, P_max_, P_min_, Tp‐e dispersion, and Tp‐e/QT ratio, were considered exploratory. Their *p*‐values are therefore presented as nominal and should be interpreted as descriptive and hypothesis‐generating.

Effect sizes are reported as Cohen's d for between‐group comparisons and, for multivariable regression models, as unstandardized regression coefficients (B) with 95% confidence intervals together with standardized β coefficients.

To directly address the potential influence of the recording‐level Holter quality criteria, a post hoc segment‐based sensitivity analysis was performed for the LF/HF ratio. Participants who had been excluded from the primary Holter analysis solely because the complete recording exceeded the prespecified artifact or non‐sinus beat threshold were reconsidered if at least one artifact‐free 5‐min segment was available within each prespecified daytime and nighttime period. LF/HF values were derived using the same spectral‐processing protocol as in the primary analysis. These participants were added to the primary cohort to form an expanded segment‐based cohort. The LF/HF ratio was compared between the obesity and normal‐weight groups using Welch's independent‐samples *t*‐test, and its association with EAT thickness within the obesity group was reassessed using Spearman rank correlation. SDNN and RMSSD were not included in this sensitivity analysis because they were calculated from the complete 24‐h normal‐to‐normal interval series.

## 3 Results

### 3.1 Study Flow and Final Cohort

A total of 624 records were screened during the study period. To maximize phenotypic separation between groups, individuals with overweight status (BMI 25.0–29.9 kg/m^2^; *n* = 142) were excluded a priori. The remaining 482 participants underwent further eligibility assessment, and 98 were excluded for predefined clinical reasons, including established cardiovascular disease, sustained arrhythmias, hypertension, diabetes mellitus, thyroid dysfunction, documented obstructive sleep apnea, smoking‐ or nicotine‐related exposure, pregnancy or lactation, electrolyte abnormalities, or use of medications known to affect autonomic function or ventricular repolarization. Of the 384 participants proceeding to data‐quality assessment, 99 were further excluded because of inadequate ECG quality for interval analysis (*n* = 20), inadequate Holter recordings for HRV analysis (*n* = 51), or inadequate echocardiographic image quality for EAT measurement (*n* = 28). The final analytic cohort therefore consisted of 285 participants, including 129 individuals with obesity and 156 normal‐weight controls. These data are summarized in the study flow chart (Figure 1).

### 3.2 Clinical Findings After Evaluation for Palpitations

Final clinical findings after evaluation for palpitations are summarized in Table S7. No clinically relevant arrhythmia was identified in 114 participants (40.0%). The remaining findings comprised isolated premature atrial contractions in 49 (17.2%), isolated premature ventricular contractions in 60 (21.1%), mixed atrial and ventricular ectopy in 27 (9.5%), sinus tachycardia in 19 (6.7%), and other documented rhythm findings in 16 (5.6%). The overall distribution of final clinical findings did not differ significantly between the normal‐weight and obesity groups (χ^2^ (Rossi et al. 2015) = 5.69; *p* = 0.338). By design, no participant with a sustained arrhythmia was included in the final analytic cohort.

### 3.3 Baseline Demographic, Echocardiographic, and Hemodynamic Characteristics

Baseline demographic, echocardiographic, hemodynamic, and metabolic characteristics are presented in Table 1. Age and sex distributions were similar between the groups, supporting reasonable comparability at the demographic level. In contrast, individuals with obesity showed a markedly less favorable adiposity‐related and metabolic profile. BMI was substantially higher in the obesity group (34.6 ± 2.7 vs. 22.5 ± 3.4 kg/m^2^, *p* < 0.001), with a very large effect size (Cohen's d = 3.88). EAT thickness was also significantly greater in the obesity group (7.2 ± 1.1 vs. 4.2 ± 2.1 mm, *p* < 0.001; Cohen's d = 1.70), indicating a clear excess of cardiac visceral adiposity.

**TABLE 1:** Demographic, echocardiographic, and hemodynamic parameters.

Variable	Controls (*n* = 156)	Individuals with obesity (*n* = 129)	Mean difference (95% CI)	Cohen's d	*t*/*χ* ^2^‐value	*p*
Age (years)	34.6 ± 6.3	33.2 ± 5.9	1.4 (−0.1 to 2.9)	0.23	1.93	0.060
Female *n* (%)	53 (33.97)	42 (32.55)	—	—	χ^2^(1) = 0.02	0.890
BMI (kg/m^2^)	22.5 ± 3.4	34.6 ± 2.7	−12.1 (−12.8 to −11.4)	−3.88	−33.48	< 0.001
EAT (mm)	4.2 ± 2.1	7.2 ± 1.1	−3.0 (−3.4 to −2.6)	−1.70	−15.46	< 0.001
SBP (mmHg)	118.0 ± 11.5	123.1 ± 14.4	−5.1 (−8.2 to −2.0)	−0.40	−3.25	0.001
DBP (mmHg)	73.2 ± 11.4	76.2 ± 13.2	−3.0 (−5.9to −0.1)	−0.24	−2.03	0.045
Fasting glucose (mg/dL)	94.2 ± 14.2	101.3 ± 21.4	−7.1 (−11.4 to −2.8)	−0.40	−3.23	0.001
LDL‐C (mg/dL)	101.4 ± 11.4	115.7 ± 21.5	−14.3 (−18.4 to −10.2)	−0.85	−6.80	< 0.001
HDL‐C (mg/dL)	55.2 ± 7.4	41.5 ± 6.3	13.7 (12.1 to 15.3)	1.98	16.88	< 0.001
TG (mg/dL)	105.3 ± 17.2	135.0 ± 33.1	−29.7 (−36.1 to −23.3)	−1.16	−9.21	< 0.001

*Note:* Values are presented as mean ± SD or percentage, as appropriate. Mean differences were calculated as controls minus individuals with obesity. Continuous variables were compared using Welch's *t*‐test, and categorical variables were compared using the chi‐square test. Effect sizes for continuous variables are presented as Cohen's d. This table is descriptive and summarizes demographic, echocardiographic, hemodynamic, and metabolic characteristics of the study groups; no multiplicity adjustment was applied to the *p*‐values reported in this table.

Abbreviations: BMI, body mass index; EAT, epicardial adipose tissue; SBP, systolic blood pressure; DBP, diastolic blood pressure; LDL‐C, low‐density lipoprotein cholesterol; HDL‐C, high‐density lipoprotein cholesterol; TG, triglycerides; CI, confidence interval.

Hemodynamic and metabolic differences showed the same overall direction. Compared with controls, individuals with obesity had higher systolic blood pressure (123.1 ± 14.4 vs. 118.0 ± 11.5 mmHg, *p* = 0.001) and slightly higher diastolic blood pressure (76.2 ± 13.2 vs. 73.2 ± 11.4 mmHg, *p* = 0.045). Fasting glucose and LDL‐C were higher in the obesity group, whereas HDL‐C was lower and triglyceride levels were higher (all *p* ≤ 0.001). Overall, Table 1 indicates that the obesity group was characterized not only by greater generalized adiposity, but also by greater epicardial fat burden and a less favorable cardiometabolic profile.

### 3.4 Autonomic and Electrophysiological Characteristics

Autonomic and electrophysiological findings are summarized in Table 2. The prespecified secondary autonomic endpoints all differed significantly between groups. Individuals with obesity had substantially lower SDNN (44.2 ± 7.3 vs. 67.3 ± 11.4 ms, *p* < 0.001), lower RMSSD (22.5 ± 2.9 vs. 36.2 ± 3.7 ms, *p* < 0.001), and a higher LF/HF ratio (3.1 ± 0.4 vs. 1.6 ± 0.3, *p* < 0.001). These findings indicate a markedly less favorable autonomic profile in the obesity group, consistent with reduced vagal modulation and relative sympathovagal imbalance. All three secondary autonomic endpoints remained significant under the prespecified Bonferroni‐adjusted threshold.

**TABLE 2:** Autonomic and electrophysiological parameters.

Variable	Controls (*n* = 156)	Individuals with obesity (*n* = 129)	Mean difference (95% CI)	Cohen's d	*t*‐value	*p*
SDNN, ms	67.3 ± 11.4	44.2 ± 7.3	23.1 (20.9 to 25.3)	2.37	20.69	< 0.001
RMSSD, ms	36.2 ± 3.7	22.5 ± 2.9	13.7 (12.9 to 14.5)	4.08	35.03	< 0.001
LF/HF	1.6 ± 0.3	3.1 ± 0.4	−1.5 (−1.58 to −1.42)	−4.30	−35.19	< 0.001
Basal ECG heart rate, bpm	71.3 ± 7.2	76.3 ± 8.2	−5.0 (−6.8 to −3.2)	−0.65	−5.41	< 0.001
QT, ms	363.3 ± 22.4	411.3 ± 24.5	−48.0 (−53.5 to −42.5)	−2.05	−17.11	< 0.001
QTc‐B, ms	396.0 ± 23.6	463.8 ± 26.8	−67.8 (−73.8 to −61.8)	−2.70	−22.43	< 0.001
QTc‐F, ms	384.8 ± 23.3	445.6 ± 25.7	−60.8 (−66.6 to −55.0)	−2.49	−20.73	< 0.001
P_max_, ms	107.4 ± 21.4	134.1 ± 23.1	−26.7 (−31.9 to −21.5)	−1.20	−10.04	< 0.001
P_min_, ms	90.1 ± 15.2	103.2 ± 17.3	−13.1 (−16.9 to −9.3)	−0.81	−6.72	< 0.001
PWD, ms	17.3 ± 2.4	30.9 ± 5.8	−13.6 (−14.7 to −12.5)	−3.13	−24.93	< 0.001
Tp‐e, ms	83.5 ± 7.4	96.2 ± 8.8	−12.7 (−14.6 to −10.8)	−1.58	−13.02	< 0.001
Tp‐eD, ms	11.2 ± 2.1	17.2 ± 3.1	−6.0 (−6.6 to −5.4)	−2.31	−18.72	< 0.001
Tp‐e/QT ratio	0.210 ± 0.02	0.215 ± 0.02	−0.005 (−0.010 to −0.0003)	−0.25	−2.10	0.037†

*Note:* Values are presented as mean ± SD. Mean differences were calculated as controls minus individuals with obesity. Continuous variables were compared using Welch's *t*‐test, and effect sizes are presented as Cohen's d. The prespecified co‐primary electrophysiological endpoints were P‐wave dispersion (PWD) and Tpeak‐to‐Tend (Tp‐e) interval; for these two endpoints, a two‐sided *p*‐value < 0.05 was considered statistically significant. The prespecified secondary autonomic endpoints were SDNN, RMSSD, and LF/HF ratio; for these three endpoints, Bonferroni correction was applied, and statistical significance was defined as *p* < 0.0167. All other electrocardiographic variables shown in this table, including basal ECG heart rate, QT, QTc‐B, QTc‐F, P_max_, P_min_, Tp‐e dispersion, and Tp‐e/QT ratio, were treated as exploratory/descriptive measures, and their *p*‐values should therefore be interpreted as nominal.

Abbreviations: ECG, electrocardiogram; SDNN, standard deviation of normal‐to‐normal intervals; RMSSD, root mean square of successive differences; LF/HF, low‐frequency‐to‐high‐frequency ratio; QTc‐B, Bazett‐corrected QT interval; QTc‐F, Fridericia‐corrected QT interval; P_max_, maximum P‐wave duration; P_min_, minimum P‐wave duration; PWD, P‐wave dispersion; Tp‐e, Tpeak‐to‐Tend interval; Tp‐eD, Tpeak‐to‐Tend dispersion.

**FIGURE 2:**
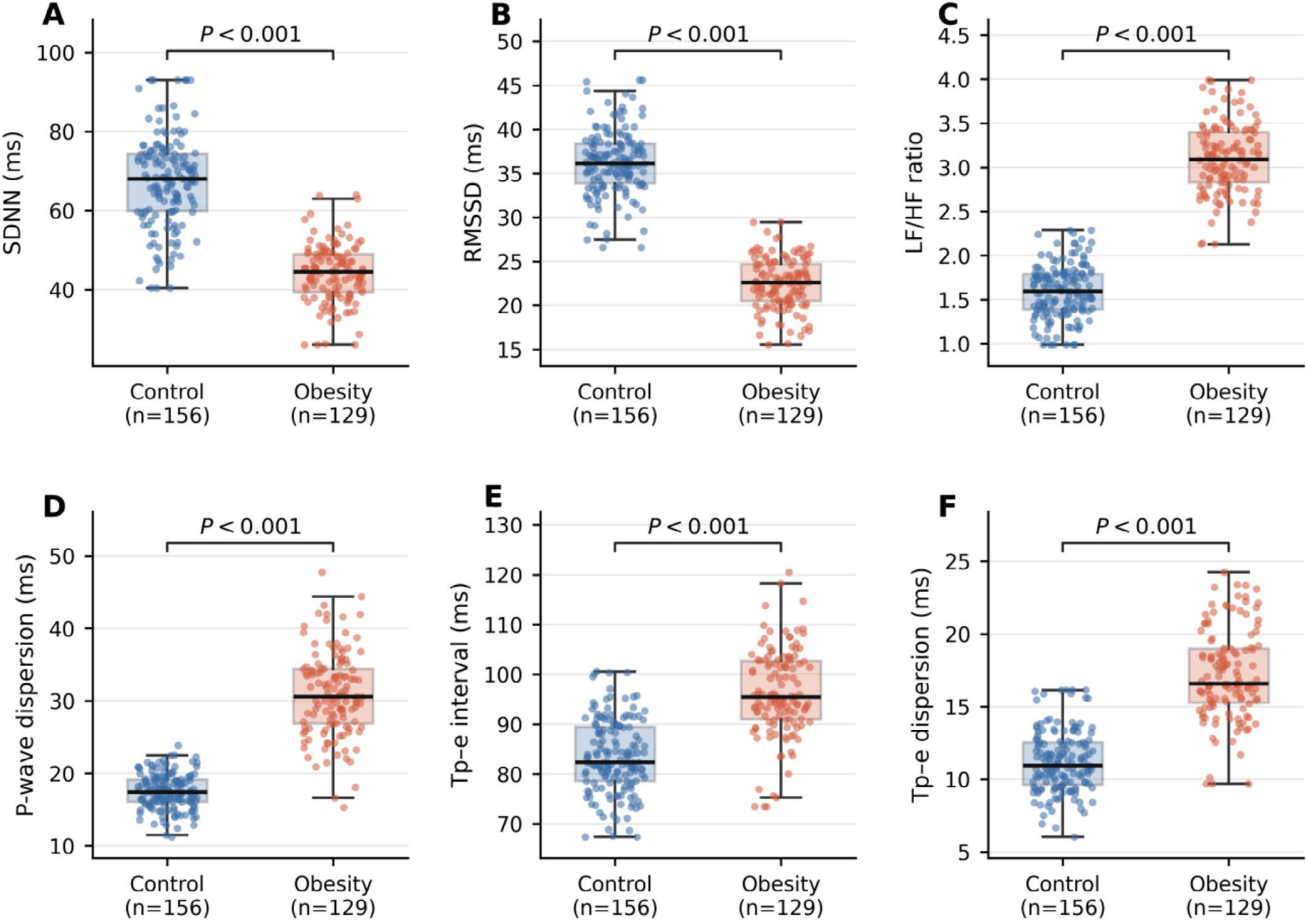
Comparison of heart rate variability and electrocardiographic indices between normal‐weight controls and individuals with obesity. Boxplots show the median and interquartile range; whiskers extend to 1.5 times the interquartile range, and overlaid dots represent individual participants. (A) SDNN, (B) RMSSD, (C) LF/HF ratio, (D) *P*‐wave dispersion, (E) Tpeak‐to‐Tend interval, and (F) Tpeak‐to‐Tend dispersion. *p* values were calculated using two‐sided Welch's independent‐samples *t* tests.

The prespecified co‐primary electrophysiological endpoints were also clearly different between groups. P‐wave dispersion was substantially higher in individuals with obesity than in controls (30.9 ± 5.8 vs. 17.5 ± 2.4 ms; mean difference, −13.6 ms; 95% CI −14.5 to −12.3; *p* < 0.001), with a very large effect size (Cohen's d = 3.13). Similarly, the Tp‐e interval was longer in the obesity group (96.2 ± 8.8 vs. 83.5 ± 7.4 ms; mean difference, −12.7 ms; 95% CI −14.6 to −10.8; *p* < 0.001), with a large effect size (Cohen's d = 1.58). Thus, both prespecified co‐primary markers supported greater electrical heterogeneity in obesity (Figure 2).

Exploratory electrocardiographic variables showed a directionally consistent pattern. Basal ECG heart rate was higher in the obesity group. QT, QTc‐B, and QTc‐F were all prolonged, and both P_max_ and P_min_ were increased. Tp‐e dispersion was also higher in individuals with obesity (17.2 ± 3.1 vs. 11.2 ± 2.1 ms, *p* < 0.001). By contrast, the Tp‐e/QT ratio showed only a nominal between‐group difference (0.215 ± 0.020 vs. 0.210 ± 0.020, *p* = 0.037), and, in keeping with the prespecified endpoint hierarchy, this finding should be interpreted as exploratory rather than confirmatory. Overall, Table 2 shows that obesity was associated with a broadly less favorable autonomic‐electrophysiological phenotype, with the most robust between‐group signals observed for SDNN, RMSSD, LF/HF ratio, PWD, and Tp‐e.

To examine potential selection bias arising from the recording‐level Holter quality criteria, participants included in the final Holter analysis were compared with those excluded because of insufficient analyzable duration, excessive artifact burden, or a non‐sinus beat burden exceeding 5% (Table S9). The two groups were broadly similar with respect to age, sex, body mass index, waist circumference, systolic and diastolic blood pressure, and resting ECG heart rate, with no statistically significant differences across the assessed baseline characteristics (all *p* ≥ 0.541).

**FIGURE 3:**
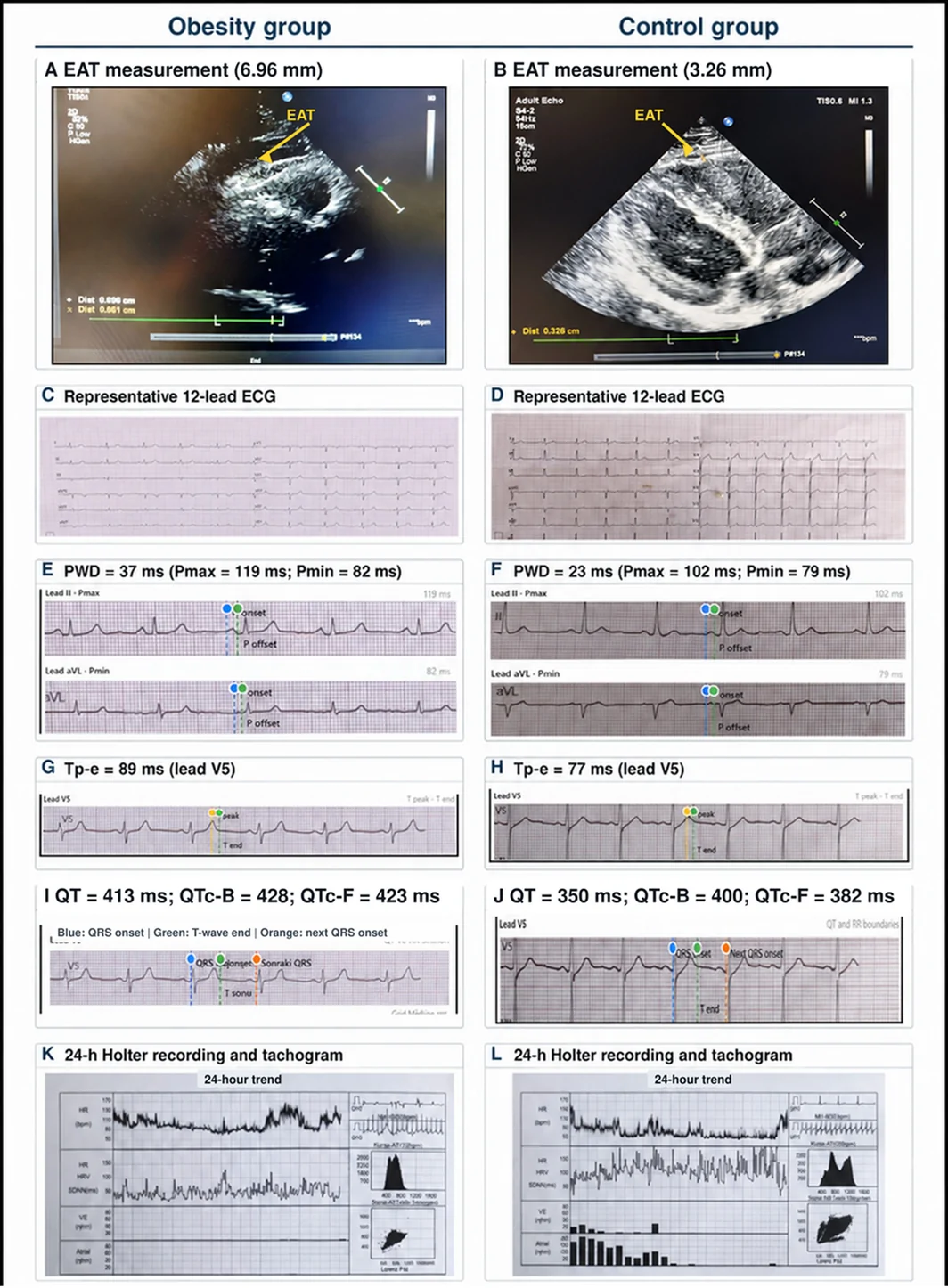
Representative echocardiographic, electrocardiographic, and Holter findings in the obesity and control groups. Panels A (obesity group) and B (control Group) show representative transthoracic echocardiographic images used for epicardial adipose tissue (EAT) thickness measurement. Panels C and D show representative 12‐lead electrocardiograms. Panels E and F illustrate P‐wave dispersion (PWD), calculated as the difference between the maximum and minimum P‐wave durations (Pmax−Pmin), in the obesity and control groups, respectively. Panels G and H demonstrate Tpeak‐to‐Tend (Tp‐e) interval measurements in lead V5. Panels I and J demonstrate QT and RR interval measurements in lead V5, with heart rate–corrected QT intervals calculated using the Bazett (QTcB) and Fridericia (QTcF) formulas. Panels K and L show representative 24‐hour Holter recordings, including heart‐rate and heart‐rate‐variability tachograms, representative ECG strips, NN/RR interval histograms, and Lorenz plots. The left column represents the obesity group, whereas the right column represents the normal‐weight control group. ECG, electrocardiogram; EAT, epicardial adipose tissue; HRV, heart rate variability; PWD, P‐wave dispersion; SDNN, standard deviation of normal‐to‐normal intervals; QTcB, Bazett‐corrected QT interval; QTcF, Fridericia‐corrected QT interval; Tp‐e, Tpeak‐to‐Tend interval.

The distributions of the principal autonomic and electrophysiological indices, including individual participant values, are presented in Figure 2. Representative echocardiographic images, 12‐lead ECG recordings with annotated PWD, Tp‐e, and QT/QTc measurements, and 24‐h Holter recordings with corresponding tachograms from both study groups are shown in Figure 3.

### 3.5 Multivariable Linear Regression Analyses for the Co‐Primary Electrical Heterogeneity Markers

Multivariable linear regression results for the two prespecified co‐primary electrophysiological endpoints are presented in Table 3. In the model with P‐wave dispersion as the dependent variable, greater EAT thickness remained independently associated with higher PWD after adjustment for age, sex, BMI, systolic blood pressure, and HDL‐C (B = 1.82, 95% CI 1.20 to 2.44; standardized β = 0.38; *p* < 0.001). Higher HDL‐C was independently associated with lower PWD (B = −0.10, 95% CI –0.17 to −0.03; β = −0.21; *p* = 0.007), whereas age, sex, BMI, and systolic blood pressure were not independently associated with PWD. Model performance was moderate, with R^2^ = 0.46 and adjusted R^2^ = 0.44.

**TABLE 3:** Multivariable linear regression models for the co‐primary electrical heterogeneity markers.

Panel A. Dependent variable: P‐wave dispersion (PWD)				
Variable	Unstandardized B (95% CI)	Standardized β	*t*	*p*
Epicardial adipose tissue thickness, per 1 mm	1.82 (1.20 to 2.44)	0.38	5.72	< 0.001
Age, per 1 year	0.08 (−0.02 to 0.18)	0.06	1.48	0.140
Male sex (vs. Female)	0.86 (−1.01 to 2.73)	0.04	0.91	0.364
Body mass index, per 1 kg/m^2^	0.26 (−0.05 to 0.53)	0.12	1.66	0.098
Systolic blood pressure, per 1 mmHg	0.04 (−0.01 to 0.09)	0.08	1.58	0.116
HDL‐C, per 1 mg/dL	−0.10 (−0.17 to −0.03)	−0.21	−2.74	0.007
Model A performance**:** R^2^ = 0.46; adjusted R^2^ = 0.44.				

Panel B. Dependent variable: Tp‐e interval				
Variable	Unstandardized B (95% CI)	Standardized β	*t*	*p*
Epicardial adipose tissue thickness, per 1 mm	2.10 (1.32 to 2.88)	0.35	5.31	< 0.001
Age, per 1 year	0.08–0.06 to 0.22	0.05	1.16	0.247
Male sex (vs. Female)	0.62 (−1.11 to 2.35)	0.03	0.72	0.472
Body mass index, per 1 kg/m^2^	0.23 (−0.04 to 0.50)	0.11	1.69	0.093
Systolic blood pressure, per 1 mmHg	0.04 (−0.01 to 0.09)	0.07	1.47	0.143
HDL‐C, per 1 mg/dL	−0.08 (−0.14 to −0.02)	−0.18	−2.41	0.017
Model B performance: R^2^ = 0.41; adjusted R^2^ = 0.39.				

*Note:* Data are presented as unstandardized regression coefficients (B) with 95% confidence intervals and standardized beta coefficients (β). Standardized β values are provided to facilitate comparison of the relative effect sizes across predictors, whereas inferential interpretation is based primarily on unstandardized B coefficients with 95% confidence intervals. Male sex was coded as 1 for male and 0 for female.

A similar pattern was observed in the model with the Tp‐e interval as the dependent variable. Greater EAT thickness remained independently associated with a longer Tp‐e interval (B = 2.10, 95% CI 1.32 to 2.88; β = 0.35; *p* < 0.001). Higher HDL‐C again showed an inverse independent association (B = −0.08, 95% CI –0.14 to −0.02; β = −0.18; *p* = 0.017), whereas age, sex, BMI, and systolic blood pressure were not independently associated with Tp‐e. This model likewise showed moderate explanatory capacity (R^2^ = 0.41; adjusted R^2^ = 0.39). Taken together, Table 3 indicates that EAT thickness showed the most consistent independent association with both prespecified co‐primary electrical heterogeneity markers, whereas generalized adiposity as captured by BMI did not retain independent significance in either primary model.

### 3.6 Supplementary Analyses

The supplementary analyses were designed to test whether the primary findings were robust across different analytic frames rather than being driven by a single modeling choice. These analyses are presented in Tables S1–S5. The overall pattern was internally consistent and supportive of the main results.

First, within the obesity group alone, greater EAT thickness remained directionally associated with a less favorable autonomic and electrophysiological profile (Table S1). Specifically, EAT thickness correlated positively with PWD (*r* = 0.31, *p* < 0.001), Tp‐e interval (ρ = 0.28, *p* = 0.003), Tp‐e dispersion (ρ = 0.24, *p* = 0.006), and LF/HF ratio (ρ = 0.20, *p* = 0.019), and negatively with SDNN (ρ = −0.22, *p* = 0.013) and RMSSD (ρ = −0.19, *p* = 0.036). Although these within‐group correlations were modest in magnitude, they are important because they show that the direction of association persisted even within a narrower obesity‐only phenotype, rather than being explained solely by the contrast between obese participants and controls.

Second, in obesity‐restricted multivariable models that additionally included heart rate as a physiological covariate (Table S2), greater EAT thickness remained independently associated with higher PWD (B = 1.11, 95% CI 0.45 to 1.77; *p* = 0.001) and a longer Tp‐e interval (B = 0.87, 95% CI 0.18 to 1.55; *p* = 0.017). EAT thickness also showed a nominal association with Tp‐e dispersion (B = 0.75, 95% CI 0.09 to 1.41; *p* = 0.028). These findings strengthen the primary analysis by showing that the central relationship between EAT and electrical heterogeneity persisted even after restricting the analysis to participants with obesity and adjusting for heart rate.

Third, in pooled‐sample sensitivity models additionally adjusted for obesity‐group status (Table S3), EAT thickness continued to show directionally consistent and nominally significant associations with PWD (B = 0.95, 95% CI 0.34 to 1.56; *p* = 0.005), Tp‐e interval (B = 0.79, 95% CI 0.17 to 1.20; *p* = 0.022), and Tp‐e dispersion (B = 0.67, 95% CI 0.03 to 1.31; *p* = 0.039). This is an important robustness check, because it suggests that the observed relationship between EAT thickness and electrical heterogeneity was not fully explained by case–control grouping itself. In other words, EAT retained signal beyond simple obesity‐status classification.

Fourth, alternative models replacing BMI with waist circumference (Table S4) yielded materially similar results. In these models, greater EAT thickness remained independently associated with PWD (B = 1.49, 95% CI 0.88 to 2.08; *p* < 0.001), Tp‐e interval (B = 1.16, 95% CI 0.55 to 1.77; *p* = 0.002), and Tp‐e dispersion (B = 0.94, 95% CI 0.29 to 1.59; *p* = 0.003). These findings support the conclusion that the primary associations were not dependent on the exclusive use of BMI as the adiposity covariate and remained directionally stable when central adiposity was modeled using waist circumference instead.

Finally, to address the possibility of selection bias related to data‐quality exclusions, included participants were compared with those excluded at the ECG, Holter, or echocardiographic quality‐control stage (Table S5). Included and excluded participants were broadly similar with respect to age, sex, BMI, waist circumference, systolic blood pressure, diastolic blood pressure, and basal ECG heart rate, with no statistically significant between‐group differences. This comparison supports the conclusion that exclusion at the data‐quality stage was unlikely to have materially distorted the baseline profile of the final analytic cohort.

Cardiac chamber size was modestly associated with the electrical heterogeneity markers (Table S8). PWD correlated with both LAVI (*r* = 0.30, *p* < 0.001) and left atrial diameter (*r* = 0.24, *p* < 0.001), whereas Tp‐e correlated with LVMI (*r* = 0.27, *p* < 0.001) and LVEDD (*r* = 0.22, *p* = 0.001). Similar correlations were observed for Tp‐eD with LVMI and LVEDD (*r* = 0.24 and r = 0.18, respectively; both *p* ≤ 0.005). After separate adjustment for LAVI or left atrial diameter, the obesity coefficient for PWD remained large (B = 12.53 and B = 12.73 ms, respectively), corresponding to attenuation of only 6.4% and 4.7%. Adjustment for LVMI or LVEDD similarly resulted in limited attenuation of the obesity coefficients for Tp‐e (6.8% and 4.2%) and Tp‐eD (6.2% and 2.8%). These findings indicate that cardiac chamber size contributed modestly to, but did not account for, the observed between‐group differences in electrical heterogeneity.

To assess potential selection bias specifically related to the recording‐level Holter quality criteria, participants included in the final Holter analysis were compared with those excluded because of insufficient analyzable duration, excessive artifact burden, or a non‐sinus beat burden exceeding 5% (Table S9). Included and excluded participants were broadly similar with respect to age, sex, body mass index, waist circumference, systolic and diastolic blood pressure, and resting ECG heart rate, with no statistically significant differences across the assessed baseline characteristics (all *p* > 0.05). These findings suggest that Holter‐related exclusions did not materially alter the measured baseline profile of the analytic cohort.

In the post hoc segment‐based sensitivity analysis, the expanded cohort comprised 336 participants, including 186 normal‐weight controls and 150 individuals with obesity. The LF/HF ratio remained markedly higher in individuals with obesity than in controls (3.13 ± 0.39 vs. 1.64 ± 0.35), corresponding to a mean difference of 1.49 (95% CI 1.41–1.57; Cohen's d = 4.04; *p* < 0.001). Within the expanded obesity group, EAT thickness remained positively correlated with the LF/HF ratio (ρ = 0.19, 95% CI 0.02–0.36; *p* = 0.021). These estimates were materially similar to those obtained in the primary analytic cohort, indicating that the principal frequency‐domain finding was robust to inclusion of recordings with usable artifact‐free 5‐min segments despite failure to satisfy the complete‐recording quality threshold (Table S10).

Overall, the supplementary analyses support the internal consistency of the main findings across within‐group correlation analysis, obesity‐restricted multivariable modeling, pooled models additionally adjusted for group status, and alternative adiposity‐modeling strategies. Accordingly, Tables S1–S10 should be interpreted not as peripheral add‐ons, but as structured robustness analyses showing that the main association between greater EAT thickness and a less favorable autonomic‐electrophysiological profile remained directionally stable across multiple analytic approaches.

## 4 Discussion

The present retrospective cross‐sectional study provides an integrated evaluation of epicardial adipose tissue, autonomic modulation, and electrocardiographic markers of electrical heterogeneity in adults with obesity. Three findings stand out. First, compared with normal‐weight controls, individuals with obesity had greater EAT thickness together with a less favorable autonomic and electrophysiological profile. Second, obesity was associated with lower HRV indices and greater atrial and ventricular electrical heterogeneity, particularly higher P‐wave dispersion and a longer Tp‐e interval. Third, in multivariable models, greater EAT thickness remained independently associated with both co‐primary electrical heterogeneity markers, whereas body mass index did not retain independent significance. Taken together, these findings support the view that obesity is accompanied by a less favorable autonomic‐electrophysiological phenotype and that EAT may represent a meaningful component of this phenotype (Grassi et al. 2019, 2020; La Rovere et al. 1998; Rossi et al. 2015; Iacobellis and Bianco 2011; Mahabadi et al. 2013; Al Chekakie et al. 2010; Batal et al. 2010; Drossos et al. 2014; Nasri et al. 2018; Dilaveris and Gialafos 2001; Goldman et al. 2023; Task Force of the European Society of Cardiology and the North American Society of Pacing and Electrophysiology 1996; Wong et al. 2011).

One of the most clinically interesting observations in the present study is that EAT thickness remained independently associated with both PWD and the Tp‐e interval even after adjustment for age, sex, systolic blood pressure, HDL‐C, and BMI. This pattern matters because it suggests that cardiac visceral adiposity may capture information not fully conveyed by generalized adiposity alone. BMI is useful for describing overall body size, but it does not reflect regional fat distribution or the local biological environment surrounding the heart. EAT, by contrast, lies in direct anatomical continuity with the myocardium and coronary vasculature and may therefore better reflect local processes involved in myocardial electrical remodeling. In that sense, the persistence of EAT in the primary models, despite the loss of independent significance for BMI, supports the possibility that EAT may help phenotype obesity‐related cardiovascular remodeling more specifically than anthropometric measures alone (Iacobellis and Bianco 2011; Mahabadi et al. 2013; Al Chekakie et al. 2010; Batal et al. 2010; Drossos et al. 2014; Nasri et al. 2018; Shamloo et al. 2019; Anagnostopoulos et al. 2023; Conte et al. 2022; Bertaso et al. 2013; Iacobellis et al. 2005).

Several biological mechanisms may underlie this association. EAT is not merely a passive energy store; it is a metabolically active tissue capable of releasing proinflammatory cytokines, adipokines, and vasoactive mediators in close proximity to the myocardium (Iacobellis and Bianco 2011; Mahabadi et al. 2013). Through paracrine and vasocrine signaling, it may contribute to myocardial inflammation, conduction inhomogeneity, interstitial fibrosis, and repolarization abnormalities (Iacobellis and Bianco 2011; Mahabadi et al. 2013; Nasri et al. 2018; Goldman et al. 2023; Patel and Haldar 2022; Conte et al. 2022). Autonomic imbalance may further amplify this substrate. Obesity has consistently been linked to sympathetic predominance and reduced parasympathetic modulation, and reduced HRV has long been associated with greater arrhythmic vulnerability (Grassi et al. 2019, 2020; La Rovere et al. 1998; Rossi et al. 2015; Task Force of the European Society of Cardiology and the North American Society of Pacing and Electrophysiology 1996; Wong et al. 2011). The coexistence in our cohort of lower SDNN and RMSSD, a higher LF/HF ratio, higher basal heart rate, and more pronounced atrial and ventricular electrical heterogeneity is therefore biologically coherent and fits well within the broader concept of an adverse autonomic‐electrophysiological milieu in obesity (Grassi et al. 2019, 2020; La Rovere et al. 1998; Rossi et al. 2015; Dilaveris and Gialafos 2001; Goldman et al. 2023; Task Force of the European Society of Cardiology and the North American Society of Pacing and Electrophysiology 1996; Wong et al. 2011; Patel and Haldar 2022).

Our findings are also broadly consistent with previous work linking epicardial or pericardial fat to atrial fibrillation, ventricular arrhythmia‐related vulnerability, and adverse myocardial characteristics (Al Chekakie et al. 2010; Batal et al. 2010; Drossos et al. 2014; Wong et al. 2011, 2017; Chen et al. 2022; Nerlekar and Ha 2025; Patel and Haldar 2022; Nakamori et al. 2018; Shamloo et al. 2019; Anagnostopoulos et al. 2023; Conte et al. 2022; Takahashi et al. 2023; Thanassoulis et al. 2010; Poggi et al. 2022). Prior studies and meta‐analyses have associated greater epicardial fat burden with atrial fibrillation prevalence, recurrence after ablation, and periatrial remodeling (Al Chekakie et al. 2010; Batal et al. 2010; Wong et al. 2011, 2017; Chen et al. 2022; Nerlekar and Ha 2025; Nakamori et al. 2018; Shamloo et al. 2019; Anagnostopoulos et al. 2023; Conte et al. 2022; Takahashi et al. 2023; Thanassoulis et al. 2010; Poggi et al. 2022). More broadly, EAT has been increasingly recognized as a marker of cardiometabolic and inflammatory risk (Iacobellis and Bianco 2011; Mahabadi et al. 2013; Goldman et al. 2023; Patel and Haldar 2022; Bertaso et al. 2013; Iacobellis et al. 2005). The present study extends that literature from a different angle. Rather than focusing on overt arrhythmic outcomes, it examines intermediate phenotypic markers—HRV and ECG‐derived indices of electrical heterogeneity—within the same analytical framework. In doing so, it suggests that greater EAT thickness is linked not only to obesity itself but also to a more adverse autonomic and electrophysiological profile within obesity (Chen et al. 2022; Wong et al. 2017; Nerlekar and Ha 2025; Patel and Haldar 2022; Nakamori et al. 2018; Bertaso et al. 2013; Iacobellis et al. 2005; Takahashi et al. 2023; Thanassoulis et al. 2010; Poggi et al. 2022).

The supplementary analyses strengthen this interpretation. Within the obesity group, greater EAT thickness remained directionally associated with higher PWD, longer Tp‐e, greater Tp‐e dispersion, lower SDNN, lower RMSSD, and higher LF/HF ratio, even though these within‐group correlations were modest in magnitude. In obesity‐restricted multivariable models that additionally adjusted for heart rate, EAT thickness remained independently associated with PWD and Tp‐e, and showed a nominal association with Tp‐e dispersion. Similar directional patterns were observed in pooled models additionally adjusted for group status and in alternative models replacing BMI with waist circumference. Taken together, these analyses suggest that the observed relationship between EAT and electrical heterogeneity was not simply an artifact of case–control separation or of a particular adiposity‐modeling strategy. At the same time, because several of these supplementary associations were nominal or borderline, they are best interpreted as supportive robustness findings rather than definitive confirmatory evidence.

From a clinical perspective, these findings should be interpreted with restraint. The present study does not establish EAT thickness as a validated clinical risk‐stratification tool. Still, these findings suggest that echocardiographic EAT thickness may contribute to the phenotypic characterization of adverse autonomic–electrophysiological remodeling in selected adults with obesity. Because EAT can be assessed during routine echocardiography, this observation may be relevant for future hypothesis‐generating research, but it should not be interpreted as evidence of clinical utility. That possibility is clinically appealing because echocardiographic EAT measurement is practical and widely accessible in routine cardiology settings. Even so, the signal observed here remains associative, not prognostic. The present data do not show that greater EAT thickness predicts arrhythmic events, nor do they show that reducing EAT modifies electrical or autonomic risk. For now, EAT thickness may help with phenotyping, but it should not yet be interpreted as a stand‐alone basis for clinical decision‐making or rhythm risk estimation (Iacobellis and Bianco 2011; Anagnostopoulos et al. 2023; Conte et al. 2022).

The supplementary analyses were consistent with the primary models and suggested that the EAT–electrical heterogeneity relationship was not solely driven by group separation or by the choice of BMI as the adiposity covariate. However, because several supplementary associations were modest and nominal, these analyses should be viewed as internal consistency checks rather than confirmatory evidence.

The exclusion of individuals with overweight status warrants specific consideration. This decision was made a priori because the study was designed to compare clearly separated normal‐weight and obesity phenotypes and thereby characterize the autonomic and electrophysiological profile associated with established obesity. However, the overweight category could have provided clinically relevant information regarding whether changes in EAT thickness, HRV indices, and electrical heterogeneity develop progressively across increasing levels of adiposity. Consequently, the present study cannot determine whether the observed abnormalities emerge gradually across the BMI spectrum or become more pronounced after the obesity threshold is reached. Future studies including normal‐weight, overweight, and obesity groups—or preferably modeling BMI and other adiposity measures continuously—are needed to examine potential dose–response relationships.

## 5 Limitations

Several limitations merit consideration. First, the retrospective cross‐sectional design precludes causal inference; therefore, the observed associations should not be interpreted as evidence that EAT directly causes autonomic or electrophysiological abnormalities. Second, this was a single‐center study conducted in a selected symptomatic outpatient population evaluated for palpitations, which limits generalizability to the broader obesity population. Third, EAT was assessed as echocardiographic thickness rather than volumetric epicardial fat burden, and more comprehensive characterization with cardiac computed tomography or magnetic resonance imaging was not available (Iacobellis and Bianco 2011; Bertaso et al. 2013). In addition, the exclusion of individuals with overweight status prevented evaluation of the intermediate adiposity phenotype, precluded assessment of a potential dose–response relationship across the full BMI spectrum, and limited the generalizability of the findings to individuals with BMI values between 25.0 and 29.9 kg/m^2^.

Fourth, residual confounding remains possible despite extensive exclusion criteria, particularly from unrecognized sleep‐disordered breathing, because systematic sleep‐study data were not available. Fifth, HRV derived from 24‐h Holter recordings does not replace dedicated autonomic function testing, and ECG intervals were measured manually, although blinded analysis and high reproducibility reduce this concern. Sixth, inflammatory biomarkers, adipokines, and oxidative stress markers were not assessed, limiting mechanistic interpretation. Finally, because heart rate was higher in the obesity group, Bazett‐corrected QT values may have been partly influenced by rate‐dependent overcorrection; therefore, Fridericia‐corrected QT values were also reported as a complementary, less rate‐sensitive measure. Future prospective studies with outcome‐based follow‐up, volumetric adiposity imaging, systematic sleep‐disordered breathing assessment, and mechanistic biomarker profiling are needed to determine whether EAT‐related autonomic and electrical alterations translate into clinically meaningful arrhythmic risk (Iacobellis and Bianco 2011; Mahabadi et al. 2013; Al Chekakie et al. 2010; Batal et al. 2010; Drossos et al. 2014; Nasri et al. 2018; Patel and Haldar 2022; Anagnostopoulos et al. 2023; Conte et al. 2022; Bertaso et al. 2013; Iacobellis et al. 2005).

Although participants included in and excluded from the Holter analysis were similar across the measured baseline characteristics, the recording‐level quality criteria may have preferentially excluded individuals with a greater ectopic burden or poorer signal quality. The segment‐based sensitivity analysis supported the robustness of the LF/HF findings; however, it could not fully eliminate selection bias and was not applicable to SDNN or RMSSD because these time‐domain indices were derived from the complete 24‐h normal‐to‐normal interval series. Therefore, residual selection bias related to unmeasured electrophysiological characteristics cannot be excluded.

## 6 Conclusion

In adults with obesity, greater epicardial adipose tissue thickness was associated with impaired autonomic modulation and more pronounced electrocardiographic markers of electrical heterogeneity. These findings suggest that EAT thickness may reflect an adverse autonomic‐electrophysiological phenotype in obesity, beyond generalized adiposity alone. However, given the retrospective cross‐sectional design and the selected symptomatic outpatient population, EAT thickness should not yet be interpreted as a validated clinical risk‐stratification tool. Further prospective studies are required to determine whether these associations translate into clinically meaningful arrhythmic outcomes.

## Author Contributions

Study conception and design: Ayhan Coşgun. Data collection: Ayhan Coşgun, Hüseyin Ören. Echocardiographic and ECG analysis: Ayhan Coşgun, Hüseyin Ören. Statistical analysis and interpretation: Ayhan Coşgun. Manuscript drafting and revision: Ayhan Coşgun. All authors reviewed and approved the final version of the manuscript.

## Funding

The authors have nothing to report.

## Conflicts of Interest

The authors declare no conflicts of interest.

## Supporting information


**Table S1:** Correlations of epicardial adipose tissue thickness with electrical and autonomic markers within the obesity group.
**Table S2:** Multivariable linear regression models for electrical heterogeneity markers within the obesity group.
**Table S3:** Pooled‐sample multivariable linear regression models additionally adjusted for group status.
**Table S4:** Alternative multivariable linear regression models replacing body mass index with waist circumference.
**Table S5:** Comparison of included and excluded participants.
**Table S6:** Intraobserver and interobserver reproducibility of echocardiographic and electrocardiographic measurements.
**Table S7:** Final clinical findings after evaluation for palpitations according to study group.
**Table S8:** Cardiac chamber size and sensitivity analyses of electrocardiographic indices.
**Table S9:** Comparison of participants included in and excluded from the 24‐h Holter analysis.
**Table S10:** Segment‐based sensitivity analysis of the LF/HF ratio after inclusion of recordings with usable artifact‐free 5‐min segments.

## Data Availability

The data supporting the findings of this study are available from the corresponding author upon reasonable request. Epicardial adipose tissue thickness was significantly greater in adults with obesity than in normal‐weight controls. Obesity was associated with impaired heart rate variability and prolonged electrocardiographic markers of electrical heterogeneity. *P*‐wave dispersion and Tpeak–end interval were significantly higher in the obesity group. Epicardial adipose tissue thickness correlated with heart rate variability indices and electrocardiographic markers. Epicardial adipose tissue thickness remained associated with P‐wave dispersion and Tpeak–end interval in multivariable analyses.
