# Supplementary material for: Association of Epicardial Adipose Tissue Thickness With Heart Rate Variability and Electrocardiographic Markers of Electrical Heterogeneity in Obesity
Source: Ann Noninvasive Electrocardiol. 2026 Sep 23;31(5):e70238. doi: 10.1111/anec.70238 (PMC13601749; doi:10.1111/anec.70238)
Supplement: Supplementary file 1 [file ANEC-31-e70238-s001.docx]

## SUPPLEMENTARY MATERIAL

**Supplementary Methods**

*Supplementary sensitivity analyses*

Several supplementary analyses were performed to assess the robustness of the primary findings. These analyses were designed to determine whether the associations between epicardial adipose tissue (EAT) thickness and markers of electrical heterogeneity were consistent within the obesity subgroup, whether they persisted after accounting for study-group status in the pooled sample, and whether the results were materially altered when waist circumference was used instead of body mass index (BMI) as the adiposity measure.

First, correlation analyses were performed within the obesity group only to evaluate whether the associations observed in the pooled sample were also present within a phenotypically narrower subgroup. Pearson or Spearman correlation analysis was used as appropriate according to variable distribution, and full correlation results are provided in Supplementary Table S1.

Second, multivariable linear regression models restricted to participants with obesity were constructed for P-wave dispersion (PWD), Tpeak-to-Tend (Tp-e) interval, and Tpeak-to-Tend dispersion (Tp-eD). These models included EAT thickness as the primary independent variable of interest and were adjusted for age, sex, BMI, systolic blood pressure, and heart rate. Heart rate was additionally included in these supplementary models because it is a physiologically relevant electrophysiological covariate that may influence repolarization-related ECG measurements and may partly reflect autonomic state. Full model coefficients are shown in Supplementary Table S2.

Third, pooled-sample multivariable regression models were additionally adjusted for study-group status (obesity vs normal-weight control) to test whether the observed associations between EAT thickness and electrical heterogeneity markers were independent of case-control grouping. These models included EAT thickness together with age, sex, systolic blood pressure, heart rate, and obesity-group status. Full results are presented in Supplementary Table S3.

Fourth, alternative multivariable models were constructed by replacing BMI with waist circumference in order to examine the robustness of the findings across different adiposity metrics and to explore whether central adiposity altered the observed associations. These models included age, sex, waist circumference, systolic blood pressure, heart rate, and EAT thickness. Full coefficients are presented in Supplementary Table S4.

All supplementary regression analyses are reported as unstandardized regression coefficients with 95% confidence intervals together with standardized beta coefficients. These analyses were intended to assess robustness and consistency of the primary findings rather than to replace the prespecified primary models.

To examine whether cardiac chamber size accounted for the observed between-group differences in electrical heterogeneity markers, post hoc structural sensitivity analyses were performed. Correlations of PWD with LAVI and left atrial diameter and of Tp-e and Tp-eD with LVMI and LVEDD were first examined using Pearson correlation analysis. Separate linear regression models were then constructed for each electrical outcome. The base model included obesity status as the independent variable, and each expanded model additionally included one prespecified cardiac structural measure. LAVI and left atrial diameter were entered in separate PWD models, whereas LVMI and LVEDD were entered separately in the Tp-e and Tp-eD models to avoid collinearity. Results are reported as unstandardized regression coefficients (B) with 95% confidence intervals. Percentage attenuation of the obesity coefficient was calculated from the unrounded coefficients as 100×(B_base−B_expanded)/B_base.

**Assessment of Cardiac Chamber Size and Structural Sensitivity Analyses**

Cardiac chamber measurements were obtained from the archived transthoracic echocardiographic examinations in accordance with the recommendations of the American Society of Echocardiography and the European Association of Cardiovascular Imaging [34]. Left atrial anteroposterior diameter was measured at end-systole in the parasternal long-axis view. Left atrial volume was calculated at end-systole using the biplane method of disks from the apical four- and two-chamber views and was indexed to body surface area to obtain the left atrial volume index (LAVI). Left ventricular end-diastolic and end-systolic diameters (LVEDD and LVESD, respectively) were obtained using two-dimensional–guided linear measurements in the parasternal long-axis view. Left ventricular mass was calculated using the ASE-recommended linear method and indexed to body surface area to derive the left ventricular mass index (LVMI). Left ventricular ejection fraction was calculated using the biplane modified Simpson method.

Post hoc structural sensitivity analyses were performed to determine whether cardiac chamber size accounted for the observed differences in electrocardiographic markers of electrical heterogeneity. Group differences in cardiac structural measurements were evaluated using Welch’s independent-samples t test. Pearson correlation analyses were used to examine the associations of P-wave dispersion (PWD) with LAVI and left atrial diameter and the associations of the Tpeak-to-Tend (Tp-e) interval and Tp-e dispersion (Tp-eD) with LVMI and LVEDD.

Separate linear regression models were subsequently constructed for each electrocardiographic outcome. In the obesity-status analyses, the base model included obesity status as the independent variable, and each expanded model additionally included one cardiac structural covariate. LAVI and left atrial diameter were entered separately in the PWD models, whereas LVMI and LVEDD were entered separately in the Tp-e and Tp-eD models. Additional sensitivity models evaluated whether the association of epicardial adipose tissue thickness with PWD and Tp-e persisted after adjustment for cardiac chamber size. These models were based on the prespecified primary model, including epicardial adipose tissue thickness, age, sex, body mass index, systolic blood pressure, and high-density lipoprotein cholesterol, and were expanded by adding the corresponding structural covariate. Structural variables were entered in separate models to minimize multicollinearity.

Results are reported as unstandardized regression coefficients (B) with 95% confidence intervals. Percentage attenuation of the obesity-status or epicardial-adipose-tissue coefficient was calculated from the unrounded estimates as 100×(B_base−B_expanded)/B_base. These post hoc analyses were exploratory, and the reported p values should therefore be interpreted as nominal.

**SUPPLEMENTARY RESULTS**

Supplementary analyses are summarized in Supplementary Tables S1–S4. Within the obesity group, greater EAT thickness showed a directionally consistent relationship with a less favorable autonomic and electrophysiological profile. Specifically, higher EAT thickness tended to coincide with greater atrial and ventricular electrical heterogeneity and with less favorable heart rate variability indices, although these within-group correlations were generally modest in magnitude (Supplementary Table S1).

“In multivariable models restricted to participants with obesity, greater EAT thickness remained independently related to higher P-wave dispersion and longer Tp-e interval after adjustment for age, sex, BMI, systolic blood pressure, and heart rate. EAT thickness also showed a nominal association with Tp-e dispersion in exploratory analysis. Overall, these obesity-restricted models suggest that the relationship between EAT thickness and electrical heterogeneity was not solely attributable to the contrast between the obesity and control groups (Supplementary Table S2).

In pooled-sample sensitivity analyses additionally adjusted for group status, EAT thickness continued to show directionally consistent and nominally significant associations with P-wave dispersion, Tp-e interval, and Tp-e dispersion. These findings support that the observed relationship between EAT thickness and electrical heterogeneity was not fully explained by obesity-group membership alone (Supplementary Table S3).

Similar patterns were observed in alternative models replacing BMI with waist circumference. In these models, EAT thickness again showed directionally consistent associations with the principal markers of electrical heterogeneity, supporting the overall robustness of the main findings across different adiposity-modeling approaches, while acknowledging that some associations were of borderline statistical significance (Supplementary Table S4).

Taken together, these supplementary analyses support the general consistency of the primary findings across within-group, pooled-sample, and alternative adiposity-modeling approaches, although these results should be interpreted as supportive rather than definitive.

***Comparison of included and excluded participants***

To assess the possibility of selection bias, basic demographic, adiposity-related, hemodynamic, and heart-rate characteristics were compared between participants included in the final analytic cohort and those excluded because of inadequate ECG, Holter, or echocardiographic data quality (Supplementary Table S5). Included and excluded participants were broadly comparable across all assessed variables, including age, sex, body mass index, waist circumference, systolic and diastolic blood pressure, and basal ECG heart rate, with no statistically significant between-group differences. These findings suggest that exclusion at the data-quality stage was unlikely to have materially distorted the baseline profile of the final study sample.

Heart rate was not included in the prespecified primary models to avoid adjustment for a variable that may partly lie on the autonomic pathway of interest, therefore, heart rate was included only in supplementary robustness analyses and not in the prespecified primary models. However, supplementary models additionally adjusting for heart rate yielded materially similar patterns of results, supporting the robustness of the observed associations.

### ****SUPPLEMENTARY TABLES****

### ****Supplementary Table S1. Correlations of epicardial adipose tissue thickness with electrical and autonomic markers within the obesity group****

| **Variable pair** | **Correlation**  **coefficient** | **95% CI** | **p value** |
| --- | --- | --- | --- |
| EAT thickness vs P-wave dispersion (PWD) | r = 0.31 | 0.14 to 0.48 | <0.001 |
| EAT thickness vs Tp-e interval | ρ = 0.28 | 0.11 to 0.44 | 0.003 |
| EAT thickness vs Tp-e dispersion | ρ = 0.24 | 0.08 to 0.40 | 0.006 |
| EAT thickness vs SDNN | ρ = -0.22 | -0.39 to -0.05 | 0.013 |
| EAT thickness vs RMSSD | ρ = -0.19 | -0.36 to -0.01 | 0.036 |
| EAT thickness vs LF/HF ratio | ρ = 0.20 | 0.03 to 0.37 | 0.019 |

**Abbreviations:** EAT, epicardial adipose tissue; PWD, P-wave dispersion; Tp-e, T-peak-to-T-end; SDNN, standard deviation of all normal-to-normal intervals; RMSSD, root mean square of successive differences; LF/HF, low-frequency/high-frequency ratio; CI, confidence interval.

**Footnote:** Correlations were examined in participants with obesity only to assess whether the associations observed in the pooled sample persisted within the obesity subgroup. Pearson correlation was used for normally distributed variables and Spearman rank correlation for non-normally distributed variables, as appropriate. Ninety-five percent confidence intervals are reported for all correlation coefficients.

**Supplementary Table S2. Multivariable linear regression models for electrical heterogeneity markers within the obesity group**

**Panel A. Dependent variable: P-wave dispersion (PWD)**

| **Variable** | **Unstandardized**  **B (95% CI)** | **Standardized**  **B** | **T value** | **p value** |
| --- | --- | --- | --- | --- |
| Epicardial adipose tissue thickness,  per 1 mm | 1.11 (0.45 to 1.77) | 0.26 | 3.30 | 0.001 |
| Age, per 1 year | 0.06 (-0.05 to 0.17) | 0.05 | 1.10 | 0.285 |
| Male sex (vs Female) | 0.70 (-1.30 to 2.70) | 0.04 | 0.77 | 0.473 |
| Body mass index, per 1 kg/m² | 0.16 (-0.09 to 0.43) | 0.12 | 1.36 | 0.199 |
| Systolic blood pressure,  per 1 mmHg | 0.07 (0.01 to 0.13) | 0.18 | 2.21 | 0.030 |
| Heart rate, per 1 beat/min | 0.10 (0.03 to 0.17) | 0.20 | 2.50 | 0.014 |

**Model statistics:** R² = 0.28 ; Adjusted R² = 0.24 ; F = 7.10 ; Model p < 0.001

**Panel B. Dependent variable: Tp-e interval**

| **Variable** | **Unstandardized B**  **(95% CI)** | **Standardized B** | **T value** | **p value** |
| --- | --- | --- | --- | --- |
| Epicardial adipose tissue thickness,  per 1 mm | 0.87 (0.18 to 1.55) | 0.23 | 2.57 | 0.017 |
| Age, per 1 year | 0.05 (-0.07 to 0.17) | 0.04 | 0.82 | 0.419 |
| Male sex (vs Female) | 1.23 (-0.94 to 3.42) | 0.07 | 1.14 | 0.274 |
| Body mass index, per 1 kg/m² | 0.15 (-0.14 to 0.41) | 0.08 | 0.99 | 0.365 |
| Systolic blood pressure,  per 1 mmHg | 0.04 (-0.01 to 0.09) | 0.11 | 1.66 | 0.105 |
| Heart rate, per 1 beat/min | 0.09 (0.01 to 0.17) | 0.16 | 2.20 | 0.031 |

**Model statistics:** R² = 0.21 ; Adjusted R² = 0.17 ; F = 4.96 ; Model p < 0.001

**Panel C. Dependent variable: Tp-e dispersion**

| **Variable** | **Unstandardized B (95% CI)** | **Standardized**  **B** | **T value** | **p value** |
| --- | --- | --- | --- | --- |
| Epicardial adipose tissue thickness, per 1 mm | 0.75 (0.09 to 1.41) | 0.22 | 2.21 | 0.028 |
| Age, per 1 year | 0.04 (-0.08 to 0.16) | 0.03 | 0.57 | 0.581 |
| Male sex (vs Female) | 1.12 (-0.96 to 3.18) | 0.09 | 1.05 | 0.280 |
| Body mass index, per 1 kg/m² | 0.12 (-0.16 to 0.40) | 0.06 | 0.85 | 0.411 |
| Systolic blood pressure, per 1 mmHg | 0.06 (0.00 to 0.12) | 0.16 | 2.0 | 0.049 |
| Heart rate, per 1 beat/min | 0.07 (0.01 to 0.15) | 0.15 | 2.00 | 0.049 |

**Model statistics:** R² = 0.18 ; Adjusted R² = 0.14 ; F = 4.12 ; Model p = 0.001

**Supplementary Table S3. Pooled-sample multivariable linear regression models additionally adjusted for group status**

**Panel A. Dependent variable: P-wave dispersion (PWD)**

| **Variable** | **Unstandardized B (95% CI)** | **Standardized B** | **T value** | **p value** |
| --- | --- | --- | --- | --- |
| Epicardial adipose tissue thickness, per 1 mm | 0.95 (0.34 to 1.56) | 0.22 | 3.04 | 0.005 |
| Age, per 1 year | 0.07 (-0.01 to 0.15) | 0.08 | 1.70 | 0.095 |
| Male sex (vs Female) | 0.82 (-0.95 to 2.59) | 0.03 | 0.96 | 0.355 |
| Systolic blood pressure, per 1 mmHg | 0.07 (0.01 to 0.13) | 0.15 | 2.30 | 0.022 |
| Heart rate, per 1 beat/min | 0.07 (0.01 to 0.13) | 0.19 | 2.51 | 0.013 |
| Obesity group (vs normal-weight control) | 2.92 (0.71 to 5.13) | 0.19 | 2.65 | 0.009 |

**Model statistics:** R² = 0.35 ; Adjusted R² = 0.33 ; F = 15.1 ; Model p < 0.001

**Panel B. Dependent variable: Tp-e interval**

| **Variable** | **Unstandardized B (95% CI)** | **Standardized B** | **T value** | **p value** |
| --- | --- | --- | --- | --- |
| Epicardial adipose tissue thickness, per 1 mm | 0.79 (0.17 to 1.42) | 0.15 | 2.45 | 0.022 |
| Age, per 1 year | 0.05 (-0.05 to 0.15) | 0.03 | 0.91 | 0.362 |
| Male sex (vs Female) | 1.12 (-0.77 to 3.00) | 0.06 | 1.15 | 0.255 |
| Systolic blood pressure, per 1 mmHg | 0.05 (0.00 to 0.10) | 0.14 | 2.01 | 0.046 |
| Heart rate, per 1 beat/min | 0.08 (0.01 to 0.15) | 0.17 | 2.24 | 0.029 |
| Obesity group (vs normal-weight control) | 2.12 (0.04 to 4.20) | 0.17 | 2.00 | 0.048 |

**Model statistics:** R² = 0.27 ; Adjusted R² = 0.24 ; F = 9.74 ; Model p < 0.001

**Panel C. Dependent variable: Tp-e dispersion**

| **Variable** | **Unstandardized B (95% CI)** | **Standardized**  **B** | **T value** | **p value** |
| --- | --- | --- | --- | --- |
| Epicardial adipose tissue thickness, per 1 mm | 0.67 (0.03 to 1.31) | 0.14 | 2.10 | 0.039 |
| Age, per 1 year | 0.04 (-0.06 to 0.14) | 0.02 | 0.67 | 0.503 |
| Male sex (vs Female) | 0.95 (-0.83 to 2.73) | 0.05 | 1.06 | 0.315 |
| Systolic blood pressure, per 1 mmHg | 0.04 (0.00 to 0.08) | 0.11 | 1.80 | 0.070 |
| Heart rate, per 1 beat/min | 0.07 (0.00 to 0.14) | 0.14 | 2.00 | 0.049 |
| Obesity group (vs normal-weight control) | 1.95 (-0.19 to 4.01) | 0.12 | 1.79 | 0.075 |

**Model statistics:** R² = 0.22 ; Adjusted R² = 0.19 ; F = 7.28 ; Model p < 0.001

**Supplementary Table S4. Alternative multivariable linear regression models replacing body mass index with waist circumference**

**Panel A. Dependent variable: P-wave dispersion (PWD)**

| **Variable** | **Unstandardized B (95% CI)** | **Standardized B** | **T value** | **p value** |
| --- | --- | --- | --- | --- |
| Epicardial adipose tissue thickness, per 1 mm | 1.49 (0.88 to 2.08) | 0.33 | 4.80 | <0.001 |
| Age, per 1 year | 0.06 (-0.02 to 0.14) | 0.06 | 1.57 | 0.126 |
| Male sex (vs Female) | 0.83 (-1.02 to 2.68) | 0.04 | 0.89 | 0.381 |
| Waist circumference, per 1 cm | 0.08 (0.01 to 0.17) | 0.14 | 2.20 | 0.030 |
| Systolic blood pressure, per 1 mmHg | 0.06 (0.01 to 0.11) | 0.14 | 2.40 | 0.018 |
| Heart rate, per 1 beat/min | 0.08 (0.02 to 0.14) | 0.17 | 2.61 | 0.010 |

**Model statistics:** R² = 0.33 ; Adjusted R² = 0.30 ; F = 13.4 ; Model p < 0.001

**Panel B. Dependent variable: Tp-e interval**

| **Variable** | **Unstandardized B (95% CI)** | **Standardized B** | **T value** | **p value** |
| --- | --- | --- | --- | --- |
| Epicardial adipose tissue thickness, per 1 mm | 1.16 (0.55 to 1.77) | 0.24 | 3.40 | 0.002 |
| Age, per 1 year | 0.04 (-0.06 to 0.14) | 0.04 | 1.11 | 0.266 |
| Male sex (vs Female) | 1.18 (-0.78 to 3.14) | 0.05 | 1.19 | 0.235 |
| Waist circumference, per 1 cm | 0.06 (0.00 to 0.12) | 0.14 | 1.86 | 0.055 |
| Systolic blood pressure, per 1 mmHg | 0.04 (0.00 to 0.08) | 0.13 | 2.11 | 0.043 |
| Heart rate, per 1 beat/min | 0.07 (0.01 to 0.13) | 0.15 | 2.22 | 0.027 |

**Model statistics:** R² = 0.26 ; Adjusted R² = 0.23 ; F = 9.10 ; Model p < 0.001

**Panel C. Dependent variable: Tp-e dispersion**

| **Variable** | **Unstandardized B**  **(95% CI)** | **Standardized B** | **T value** | **p value** |
| --- | --- | --- | --- | --- |
| Epicardial adipose tissue thickness, per 1 mm | 0.94 (0.29 to 1.59) | 0.22 | 2.95 | 0.003 |
| Age, per 1 year | 0.03 (-0.06 to 0.12) | 0.02 | 0.69 | 0.493 |
| Male sex (vs Female) | 1.05 (-0.91 to 3.01) | 0.04 | 1.07 | 0.291 |
| Waist circumference, per 1 cm | 0.05 (-0.01 to 0.11) | 0.11 | 1.70 | 0.093 |
| Systolic blood pressure, per 1 mmHg | 0.04 (0.00 to 0.08) | 0.10 | 1.86 | 0.064 |
| Heart rate, per 1 beat/min | 0.06 (0.00 to 0.12) | 0.13 | 2.00 | 0.047 |

**Model statistics:** R² = 0.22 ; Adjusted R² = 0.19 ; F = 7.41 ; Model p < 0.001

**Supplementary Table S5. Comparison of included and excluded participants**

| **Variable** | **Included participants (n=285)** | **Excluded participants (n=99)** | **Mean difference / effect estimate (95% CI)** | **p value** |
| --- | --- | --- | --- | --- |
| Age, years | 34.0 ± 6.1 | 34.8 ± 6.6 | -0.8 (-2.3 to 0.7) | 0.29 |
| Female, n (%) | 95 (33.3) | 31 (31.3) | — | 0.72 |
| Male, n (%) | 190 (66.7) | 68 (68.7) | — | 0.72 |
| Body mass index, kg/m² | 28.0 ± 6.8 | 28.9 ± 7.2 | -0.9 (-2.5 to 0.7) | 0.27 |
| Waist circumference, cm | 98.6 ± 16.4 | 100.9 ± 17.2 | -2.3 (-6.1 to 1.5) | 0.23 |
| Systolic blood pressure, mmHg | 120.3 ± 13.4 | 121.9 ± 14.1 | -1.6 (-4.9 to 1.7) | 0.34 |
| Diastolic blood pressure, mm Hg | 74.6±12.3 | 75.6 ± 14.5 | -1.0 (−4.2 to 2.2) | 0.54 |
| Basal ECG heart rate, bpm | 73.6 ± 8.1 | 74.8 ± 8.7 | -1.2 (-3.2 to 0.8) | 0.24 |

**Footnote**: Excluded participants in this comparison refers only to individuals excluded at the data-quality stage because of inadequate ECG, Holter, or echocardiographic recordings.

**Supplementary Table S6. Intraobserver and interobserver reproducibility of echocardiographic and electrocardiographic measurements**

| **Parameter** | **Unit** | **Intraobserver ICC (95% CI)** | **Interobserver ICC (95% CI)** |
| --- | --- | --- | --- |
| EAT thickness | mm | 0.92 (0.88–0.95) | 0.90 (0.85–0.94) |
| Pmax | ms | 0.90 (0.82–0.98) | 0.88 (0.80–0.96) |
| Pmin | ms | 0.86 (0.72–0.90) | 0.83 (0.72–0.94) |
| PWD | ms | 0.84 (0.70–0.93) | 0.78 (0.62–0.86) |
| QT interval | ms | 0.94 (0.88–0.97) | 0.90 (0.82–0.98) |
| QTc-B | ms | 0.88 (0.78–0.98) | 0.86 (0.76–0.95) |
| QTc-F | ms | 0.87 (0.76–0.96) | 0.85 (0.77–0.96) |
| Tp-e interval | ms | 0.90 (0.84–0.96) | 0.84 (0.76–0.92) |
| Tp-eD | ms | 0.82 (0.70–0.94) | 0.78 (0.64–0.92) |
| Tp-e/QT ratio | — | 0.89 (0.80–0.97) | 0.85 (0.79–0.92) |

**Footnote**: Values are presented as intraclass correlation coefficients with 95% confidence intervals. ICCs were calculated in a randomly selected subset of 30 participants using two-way random-effects, absolute-agreement, single-measurement models. For derived parameters (PWD, QTc-B, QTc-F, Tp-eD, and Tp-e/QT), ICCs were calculated from values derived separately from each observer’s measurements before consensus adjudication; the 5-ms consensus criterion was not applied separately to these derived parameters. Basal ECG heart rate was not included in the reproducibility analysis because it was automatically generated by the digital ECG recording system rather than manually measured. Intraobserver ICCs were calculated from measurements repeated by the same observer after a two-week interval, whereas interobserver ICCs were calculated from independent measurements performed by a second blinded observer. All ICCs were derived from paired measurements obtained before consensus adjudication. HRV indices, including SDNN, RMSSD, and the LF/HF ratio, were not included in the observer reproducibility analysis because they were generated automatically by the Holter analysis software after standardized signal editing rather than being manually measured.

**Abbreviations**: CI, confidence interval; EAT, epicardial adipose tissue; ICC, intraclass correlation coefficient; PWD, P-wave dispersion; QTc-B, Bazett-corrected QT interval; QTc-F, Fridericia-corrected QT interval; Tp-e, Tpeak–Tend interval; Tp-eD, Tpeak–Tend dispersion.

**Supplementary Table S7. Final clinical findings after evaluation for palpitations according to study group**

| **Final clinical finding** | **Normal-weight controls (n=156)** | **Individuals with obesity (n=129)** | **Total (n=285)** |
| --- | --- | --- | --- |
| No clinically relevant arrhythmia identified | 71 (45.5) | 43 (33.3) | 114 (40.0) |
| Isolated premature atrial contractions | 27 (17.3) | 22 (17.1) | 49 (17.2) |
| Isolated premature ventricular contractions | 30 (19.2) | 30 (23.3) | 60 (21.1) |
| Mixed atrial and ventricular ectopy | 12 (7.7) | 15 (11.6) | 27 (9.5) |
| Sinus tachycardia | 8 (5.1) | 11 (8.5) | 19 (6.7) |
| Other documented rhythm findings | 8 (5.1) | 8 (6.2) | 16 (5.6) |
| Total | 156 (100) | 129 (100) | 285 (100) |

Global P=0.338

**Footnote:** Values are presented as n (%). For this post hoc descriptive analysis, final clinical findings were retrospectively abstracted from the cardiology assessment completed after resting electrocardiography, transthoracic echocardiography, and 24-hour Holter monitoring. Diagnostic categories were mutually exclusive. Participants with both atrial and ventricular ectopy were classified as having mixed ectopy, whereas the isolated premature atrial and ventricular contraction categories were used when only one type of ectopy was documented. “Other documented rhythm findings” comprised sinus arrhythmia in 7 participants (normal-weight controls, n=4; individuals with obesity, n=3), brief nonsustained atrial runs in 5 participants (normal-weight controls, n=2; individuals with obesity, n=3), and first-degree or Mobitz type I second-degree atrioventricular block in 4 participants (normal-weight controls, n=2; individuals with obesity, n=2). No Mobitz type II second-degree or third-degree atrioventricular block was identified. The overall distribution of clinical findings was compared using the Pearson chi-square test (χ²[5]=5.69; p=0.338). By design, no participant with a sustained arrhythmia was included in the final analytic cohort.

### Supplementary Table S8. Cardiac chamber size and sensitivity analyses of electrocardiographic indices

#### A. Group comparisons

| **Parameter** | **Normal-weight controls (n=156)** | **Individuals with obesity (n=129)** | **Difference: control−obesity (95% CI)** | **P value** | **Cohen’s d** |
| --- | --- | --- | --- | --- | --- |
| SDNN, ms | 67.30±11.40 | 44.20±7.30 | 23.10 (20.90 to 25.30) | <0.001 | 2.37 |
| RMSSD, ms | 36.20±3.70 | 22.50±2.90 | 13.70 (12.93 to 14.47) | <0.001 | 4.08 |
| LF/HF ratio | 1.60±0.30 | 3.10±0.40 | −1.50 (−1.58 to −1.42) | <0.001 | −4.30 |
| PWD, ms | 17.30±2.40 | 30.90±5.80 | −13.60 (−14.48 to −12.32) | <0.001 | −3.13 |
| Tp-e, ms | 83.50±7.40 | 96.20±8.80 | −12.70 (−14.62 to −10.78) | <0.001 | −1.58 |
| Tp-eD, ms | 11.20±2.10 | 17.20±3.10 | −6.00 (−6.63 to −5.37) | <0.001 | −2.31 |
| LA diameter, mm | 33.5±3.3 | 36.1±3.7 | −2.60 (−3.43 to −1.77) | <0.001 | −0.75 |
| LAVI, mL/m² | 25.3±4.0 | 29.0±5.1 | −3.70 (−4.79 to −2.61) | <0.001 | −0.82 |
| LVEDD, mm | 46.3±3.8 | 48.4±4.1 | −2.10 (−3.03 to −1.17) | <0.001 | −0.53 |
| LVESD, mm | 29.3±3.1 | 30.1±3.1 | −0.80 (−1.53 to −0.07) | 0.031 | −0.26 |
| LVMI, g/m² | 78.3±13.7 | 88.0±16.2 | −9.70 (−13.24 to −6.16) | <0.001 | −0.65 |
| LVEF, % | 63.2±3.7 | 62.6±4.1 | 0.60 (−0.32 to 1.52) | 0.200 | 0.15 |

**Footnote:** Values are presented as mean±standard deviation. Mean differences were calculated as normal-weight controls minus individuals with obesity. Continuous variables were compared using Welch’s independent-samples t test. Cohen’s d values were calculated in the same direction as the mean differences; positive values indicate higher levels in normal-weight controls, whereas negative values indicate lower levels in normal-weight controls. CI, confidence interval.

**Abbreviations**: CI, confidence interval; HF, high-frequency power; LA, left atrial; LAVI, left atrial volume index; LF, low-frequency power; LVEDD, left ventricular end-diastolic diameter; LVEF, left ventricular ejection fraction; LVESD, left ventricular end-systolic diameter; LVMI, left ventricular mass index; PWD, P-wave dispersion; RMSSD, root mean square of successive differences; SDNN, standard deviation of normal-to-normal intervals; Tp-e, Tpeak-to-Tend interval; Tp-eD, Tpeak-to-Tend dispersion.

#### B. Correlations with cardiac chamber size

| **ECG parameter** | **Structural parameter** | **Pearson r (95% CI)** | **P value** |
| --- | --- | --- | --- |
| PWD | LAVI | 0.30 (0.20–0.40) | <0.001 |
| PWD | LA diameter | 0.24 (0.14–0.35) | <0.001 |
| Tp-e | LVMI | 0.27 (0.16–0.38) | <0.001 |
| Tp-e | LVEDD | 0.22 (0.09–0.35) | 0.001 |
| Tp-eD | LVMI | 0.24 (0.14–0.34) | <0.001 |
| Tp-eD | LVEDD | 0.18 (0.06–0.29) | 0.005 |

**Abbreviations:** CI, confidence interval; LA, left atrial; LAVI, left atrial volume index; LVEDD, left ventricular end-diastolic diameter; LVMI, left ventricular mass index; PWD, P-wave dispersion; Tp-e, Tpeak-to-Tend interval; Tp-eD, Tpeak-to-Tend dispersion.

#### C. Sensitivity analyses of obesity status additionally adjusted for cardiac chamber size

| **Outcome** | **Structural covariate** | **Base-model Obesity status B (95% CI)** | **Expanded-model Obesity status B (95% CI)** | **Structural covariate B (95% CI)** | **P value for structural covariate** | **Attenuation of Obesity status B** |
| --- | --- | --- | --- | --- | --- | --- |
| PWD, ms | LAVI, per 5 mL/m² | 13.40 (12.32–14.48) | 12.53 (11.42–13.63) | 1.12 (0.61–1.65) | <0.001 | 6.4% |
| PWD, ms | LA diameter, per 5 mm | 13.60 (12.52–14.68) | 12.73 (11.64–13.83) | 1.33 (0.51–2.22) | 0.002 | 4.7% |
| Tp-e, ms | LVMI, per 10 g/m² | 12.70 (10.78–14.62) | 11.81 (9.91–13.73) | 1.01 (0.40–1.63) | 0.001 | 6.8% |
| Tp-e, ms | LVEDD, per 5 mm | 12.70 (10.78–14.62) | 12.14 (10.20–14.09) | 1.27 (0.31–2.23) | 0.010 | 4.2% |
| Tp-eD, ms | LVMI, per 10 g/m² | 6.00 (5.37–6.63) | 5.61 (4.96–6.26) | 0.43 (0.20–0.66) | <0.001 | 6.2% |
| Tp-eD, ms | LVEDD, per 5 mm | 6.00 (5.37–6.63) | 5.81 (5.18–6.45) | 0.41 (0.09–0.72) | 0.011 | 2.8% |

**Footnote:** Data are presented as unstandardized linear regression coefficients (B) with 95% confidence intervals. Obesity status was coded as 1 for individuals with obesity and 0 for normal-weight controls; therefore, positive coefficients indicate higher values of the corresponding electrocardiographic outcome in the obesity group. The base models included obesity status as the sole independent variable. Each expanded model additionally included one cardiac structural covariate. LAVI and left atrial diameter were entered separately in the PWD models, whereas LVMI and LVEDD were entered separately in the Tp-e and Tp-eD models to minimize multicollinearity. Structural covariate coefficients correspond to the increments specified in the table. Percentage attenuation of the obesity-status coefficient was calculated from the unrounded estimates as 100×(B_base−B_expanded)/B_base; consequently, the reported percentages may not be exactly reproducible from the rounded coefficients. These post hoc sensitivity analyses were exploratory, and the reported p values should be interpreted as nominal.

**Abbreviations:** CI, confidence interval; LA, left atrial; LAVI, left atrial volume index; LVEDD, left ventricular end-diastolic diameter; LVMI, left ventricular mass index; PWD, P-wave dispersion; Tp-e, Tpeak-to-Tend interval; Tp-eD, Tpeak-to-Tend dispersion.

#### D. Sensitivity models of EAT additionally adjusted for cardiac chamber size

| **Outcome** | **Structural covariate** | **Base-model EAT B (95% CI)** | **Expanded-model EAT B (95% CI)** | **P value for EAT in the expanded model** | **Structural covariate B (95% CI)** | **P value for structural covariate** | **Attenuation of EAT B** |
| --- | --- | --- | --- | --- | --- | --- | --- |
| PWD, ms | LAVI, per 5 mL/m² | 1.81 (1.20–2.42) | 1.67 (1.06–2.29) | <0.001 | 0.71 (0.29–1.13) | 0.001 | 7.0% |
| PWD, ms | LA diameter, per 5 mm | 1.80 (1.19–2.41) | 1.72 (1.11–2.33) | <0.001 | 0.90 (0.16–1.64) | 0.015 | 4.8% |
| Tp-e, ms | LVMI, per 10 g/m² | 2.08 (1.30–2.86) | 1.95 (1.19–2.71) | <0.001 | 0.61 (0.21–1.01) | 0.005 | 6.8% |
| Tp-e, ms | LVEDD, per 5 mm | 2.08 (1.30–2.86) | 2.00 (1.23–2.77) | <0.001 | 0.76 (0.10–1.42) | 0.029 | 4.4% |

**Footnote:** Data are presented as unstandardized regression coefficients (B) with 95% confidence intervals. P values for EAT refer to the EAT coefficient in the expanded models. Base models included EAT thickness, age, sex, body mass index, systolic blood pressure, and HDL-C. Expanded models additionally included the specified cardiac structural covariate. Structural covariates were entered in separate models to minimize multicollinearity. Percentage attenuation of the EAT coefficient was calculated from the unrounded coefficients as 100×(B_base−B_expanded)/B_base; therefore, the reported percentages may not be exactly reproducible from the rounded coefficients presented in the table. These post hoc sensitivity analyses were exploratory, and the reported P values should be interpreted as nominal.

**Abbreviations**: CI, confidence interval; EAT, epicardial adipose tissue; LA, left atrial; LAVI, left atrial volume index; LVEDD, left ventricular end-diastolic diameter; LVMI, left ventricular mass index; PWD, P-wave dispersion; Tp-e, Tpeak-to-Tend interval.

### Supplementary Table S9. Comparison of participants included in and excluded from the 24-hour Holter analysis

| **Variable** | **Included participants (n=285)** | **Excluded participants (n=51)** | **Mean difference or effect estimate (95% CI)** | **P value** |
| --- | --- | --- | --- | --- |
| Age, years | 34.0 ± 6.1 | 34.4 ± 6.3 | −0.4 (−2.30 to 1.50) | 0.676 |
| Female sex, n (%) | 95 (33.3) | 16 (31.3) | 1.09 (0.58–2.08) | 0.784 |
| Body mass index, kg/m² | 28.0 ± 6.8 | 28.3 ± 7.0 | −0.3 (−2.41 to 1.81) | 0.778 |
| Waist circumference, cm | 98.6 ± 16.4 | 100.1 ± 17.0 | −1.5 (−6.63 to 3.63) | 0.562 |
| Systolic blood pressure, mmHg | 120.3 ± 13.4 | 121.6 ± 14.0 | −1.3 (−5.52 to 2.92) | 0.541 |
| Diastolic blood pressure, mmHg | 74.6± 12.3 | 75.7 ± 14.2 | −1.1 (−4.69 to 2.89) | 0.653 |
| Resting ECG heart rate, bpm | 73.6 ± 8.1 | 74.1 ± 8.7 | −0.5 (−3.11 to 2.11) | 0.704 |

**Footnote:** Values are presented as mean ± standard deviation or n (%). For continuous variables, mean differences were calculated as included minus excluded, with 95% confidence intervals obtained using Welch’s independent-samples t test. For female sex, the effect estimate is the odds ratio comparing included with excluded participants, and the P value was calculated using the Pearson chi-square test. Excluded participants were those removed at the Holter data-quality stage because the recordings did not satisfy the prespecified requirements for analyzable duration, signal quality, or non-sinus beat burden. All 51 excluded participants had usable artifact-free 5-minute segments within both prespecified daytime and nighttime periods and were therefore included in the expanded segment-based sensitivity analysis of the LF/HF ratio.

**Abbr**:CI, confidence interval; ECG, electrocardiogram; OR, odds ratio.

**Supplementary Table S10. Segment-based sensitivity analysis of the LF/HF ratio after inclusion of recordings with usable artifact-free 5-minute segments**

| **Parameter** | **Primary analytic cohort** | **Expanded segment-based sensitivity cohort** |
| --- | --- | --- |
| Total participants, n | 285 | 336 |
| Normal-weight controls, n | 156 | 186 |
| Individuals with obesity, n | 129 | 150 |
| Normal-weight controls, LF/HF ratio | 1.6 ± 0.3 | 1.64±0.35 |
| Individuals with obesity, LF/HF ratio | 3.1 ± 0.4 | 3.13±0.39 |
| Mean difference: obesity − control (95% CI) | 1.50 (1.42–1.58) | 1.49 (1.40-1.58) |
| Cohen’s d | 4.30 | 4.04 |
| P value for between-group comparison | <0.001 | <0.001 |
| Correlation between EAT thickness and LF/HF ratio within the obesity group, ρ (95% CI) | 0.20 (0.03–0.37) | 0.19 (0.02-0.36) |
| P value for correlation | 0.019 | 0.021 |

**Footnote:** Values are presented as mean ± standard deviation unless otherwise indicated. The primary analytic cohort included only participants whose complete 24-hour Holter recordings satisfied the prespecified recording-level quality criteria. The expanded sensitivity cohort additionally included participants whose recordings failed the global artifact or non-sinus beat threshold but contained at least one artifact-free 5-minute segment within each prespecified daytime and nighttime period. Only the frequency-domain LF/HF ratio was reassessed in this analysis because it was derived from selected 5-minute segments. SDNN and RMSSD were not recalculated because these time-domain indices were derived from the complete 24-hour normal-to-normal interval series. Between-group comparisons were performed using Welch’s independent-samples t test. EAT–LF/HF associations within the obesity group were evaluated using Spearman rank correlation.

**Abbreviations**: CI, confidence interval; EAT, epicardial adipose tissue; HF, high-frequency power; LF, low-frequency power; RMSSD, root mean square of successive differences; SDNN, standard deviation of normal-to-normal intervals.
